# Highly Efficient Biodegradation of Postconsumer Polylactic Acid Waste: The First Report on *Priestia aryabhattai* SNRUSAC3 and a Newly Isolated *Bacillus* sp. SNRUSAC1

**DOI:** 10.1155/ijm/8029640

**Published:** 2026-01-07

**Authors:** Suwapha Sawiphak, Aroon Wongjiratthiti

**Affiliations:** ^1^ Program of Biology, Faculty of Science and Technology, Sakon Nakhon Rajabhat University, Sakon Nakhon, Thailand

**Keywords:** biodegradation, optimization, plastic waste, polylactic acid–degrading bacteria

## Abstract

The increasing use of polylactic acid (PLA) for single‐use packaging has led to a growing accumulation of bioplastic waste. This study presents a comprehensive approach for enhancing the degradation of postconsumer PLA packaging waste, beginning with the isolation, screening, and identification of highly effective bacteria and culminating in the statistical optimization of their specific nutritional requirements. From compost samples, two highly effective strains were identified as *Bacillus* sp. SNRUSAC1 and *Priestia aryabhattai* SNRUSAC3 based on morphological, biochemical, and 16S rDNA sequence analyses. Notably, this is the first report of PLA degradation by the species *P*. *aryabhattai*. Initially, these strains achieved approximately 13% PLA dry weight loss after 56 days. To enhance their efficiency, a statistical optimization of nutritional components was performed. Under the optimized conditions, the degradation efficiency was dramatically enhanced, with SNRUSAC1 and SNRUSAC3 achieving 62.06% and 57.61% dry weight loss, respectively, in only 30 days. This represents over a fourfold increase in degradation in approximately half the time. This optimization also revealed novel, strain‐specific requirements, with ferrous sulfate identified as a critical factor that had not been previously reported to influence the growth and degradative activity of *P*. *aryabhattai*. These findings establish *Bacillus* sp. SNRUSAC1 and *P*. *aryabhattai* SNRUSAC3 as novel, highly efficient candidates for the biodegradation of PLA plastic waste.

## 1. Introduction

The COVID‐19 pandemic significantly shifted consumer behavior toward online platforms for ordering goods and food [1–3]. Most of these products are packaged in single‐use plastic materials, which are typically excluded from circular economy systems and are rarely reused or recycled [1]. This has resulted in a substantial increase in plastic waste generation. The demand for food delivery services led to a 1.35‐fold increase in plastic packaging waste in Osaka, Japan [2]. This trend was also evident in China, where online food delivery services generated approximately 847 kilotons of plastic waste in 2021 [3]. This increase in single‐use plastic consumption has driven the search for more sustainable alternatives to conventional petroleum‐based plastics (e.g., polyethylene [PE], polypropylene [PP], and polystyrene [PS]).

Polylactic acid (PLA), a biobased plastic, is a popular sustainable packaging material due to its desirable characteristics. Its biocompatibility, biodegradability, favorable mechanical properties, and processability make its performance comparable to conventional petroleum‐based plastics [4, 5]. Furthermore, PLA degrades within several months to 2 years, significantly faster than petroleum‐based plastics, which persist for 500–1000 years [4]. Although PLA is positioned as a sustainable alternative, the widespread use of its packaging has led to accumulation rates that exceed its degradation capacity, intensifying long‐term environmental, health, and economic concerns.

The degradation of plastics, including PLA, is influenced by various abiotic and biotic factors, such as polymer type, packaging size and shape, surface area, temperature, humidity, and exposure to sunlight [6]. PLA degradation involves the breakdown of ester bonds between lactic acid monomers through microbial [7, 8], enzymatic [9, 10], photolytic [11], and hydrolytic [12] degradation. Of these, microbial degradation is considered the most crucial mechanism for PLA degradation [13] due to its ability to convert PLA into nontoxic end products such as CO_2_, H_2_O, CH_4_, and biomass under appropriate biological conditions, which helps minimize environmental pollution [14, 15]. Consequently, several studies have focused on isolating and screening efficient PLA‐degrading microorganisms as a sustainable waste management strategy [16–20].

A two‐step statistical approach was employed to optimize the culture conditions. First, the Plackett–Burman design (PBD), an efficient screening method, was used to identify key factors that significantly influence microbial processes [21]. Subsequently, response surface methodology (RSM) with a Box–Behnken design (BBD) determined the optimal levels of these significant factors [22]. This combined approach is effective for optimizing nutrient compositions [23] and enhancing PLA degradation efficiency [24], particularly in microbial PLA degradation processes [19, 20].

Therefore, this study was aimed at developing an effective microbial approach for degrading PLA plastic waste. Potent microorganisms were isolated from preenriched, microbially rich environments (i.e., compost and waste dump soil), screened, and identified via morphological, biochemical, and 16S rDNA analyses. A subsequent two‐step statistical optimization, using a PBD followed by RSM with a BBD, was employed to determine and optimize strain‐specific nutritional requirements for maximal degradation. This work provides an effective biodegradation strategy for sustainable PLA waste management.

## 2. Materials and Methods

### 2.1. Isolation of PLA‐Degrading Microorganisms

To enrich for PLA‐degrading microorganisms, 2 g of sterile PLA pellets (2003D grade, melting temperature 210°C, average molecular weight 200,000 g/mol) from NatureWorks LLC (United States) was buried in compost and waste dump soil for 30 days. Collected samples were then enriched for five successive cycles in basal medium (BM) supplemented with 1% (*v*/*v*) PLA emulsion, incubating at 37°C with 150 rpm for 7 days per cycle. The BM contained the following (per liter): 4 g ammonium sulfate ((NH_4_)_2_SO_4_), 2 g dipotassium phosphate (K_2_HPO_4_), 2 g potassium dihydrogen phosphate (KH_2_PO_4_), and 0.5 g magnesium sulfate heptahydrate (MgSO_4_·7H_2_O). The final pH was adjusted to 7.0. The PLA emulsion was prepared by dissolving 1 g of PLA in 40 mL dichloromethane, adding 10 mL of this solution to 250 mL of BM with 25 *μ*L of Tween 20, and sonicating for 5 min (VC 750, Sonics & Materials Inc., United States). Serial dilutions of the enriched cultures were plated onto PLA‐emulsified agar and incubated at 37°C for 14 days [20]. Colonies forming clear zones were selected and purified by repeated streaking on Nutrient Agar (Himedia, India) plates.

### 2.2. Screening of PLA Plastic Waste–Degrading Microorganisms

The preparation of microbial cultures and sterile PLA substrate followed the method of Sawiphak and Wongjiratthiti [19]. Each isolate was cultured in 100 mL of Nutrient Broth (Himedia, India) at 37°C for 24 h with 150 rpm agitation. Cells were harvested by centrifugation (5635 × g, 10 min, 4°C), washed twice with sterile distilled water, and resuspended to an optical density at 600 nm (OD_600_) of 1.0. The PLA substrate was prepared from postconsumer drink bags (Café Amazon, Thailand). This material, selected as a representative example of real‐world PLA‐based waste, is a PLA‐based composite material and not neat PLA. The material was then cut into standardized 1 × 1 cm pieces, hereafter referred to as “PLA coupons,” which were washed with distilled water, sterilized with 70% ethanol, and air‐dried under sterile conditions.

Isolates were screened based on three criteria: growth in BM with PLA plastic waste as the sole carbon source [25], percentage weight loss of the waste [10], and surface morphology changes [26]. For the assay, each isolate was inoculated (10% *v*/*v*, OD_600_ = 1.0) into 100 mL of BM containing 2 g of sterile PLA coupons (ca. 350 pieces), which were added individually to prevent initial clumping. The flasks were then incubated for 56 days at 37°C with continuous agitation (150 rpm) to ensure adequate dispersal and aeration while also helping to minimize potential overlapping. Microbial growth (OD_600_) was monitored weekly [19]. Samples exhibiting high turbidity were serially diluted with the corresponding sterile medium to ensure the OD_600_ reading was within the instrument′s linear range. The final OD_600_ was then calculated by multiplying the reading by the dilution factor. After incubation, the coupons were retrieved, washed with distilled water, and dried at 60°C to a constant weight. The dry weight loss was calculated [27], and surface degradation was examined by scanning electron microscopy (SEM) at 5000× magnification [28]. All experiments were conducted in triplicate. The abiotic control, containing BM and PLA coupons but no microbial inoculum, was also included under identical conditions. The final OD_600_ and weight loss data were analyzed by one‐way ANOVA with Tukey′s HSD test (SPSS v. 29.0). Isolates exhibiting the highest growth, greatest percentage weight loss, and most extensive surface changes were selected for further study.

The selected isolates were subsequently assessed for qualitative enzyme activity. Esterase/lipase activity was tested using Tributyrin Agar [29]. Protease activity was determined using Skim Milk Agar [30]. A 2 *μ*L aliquot of each isolate′s standardized cell suspension (OD_600_ = 1.0), prepared as described above, was spotted onto each medium. The plates were incubated at 37°C for 2 days and observed for the formation of a clear hydrolysis zone.

### 2.3. Identification of Selected Microorganisms

Isolates were identified via morphological, biochemical, and molecular analyses. Morphological characteristics (colony and cell) were determined by the Thailand Institute of Scientific and Technological Research (TISTR). Biochemical profiles were determined using the VITEK 2 Compact system (bioMérieux, France), also by TISTR [31]. For molecular identification, performed by the Thailand Bioresource Research Center (TBRC), genomic DNA was extracted using the Genomic DNA Mini Kit (Geneaid, Taiwan). The 16S rRNA gene was amplified by PCR using primers 20F (5 ^′^–GAG TTT GAT CCT GGC TCA G–3 ^′^) and 1500R (5 ^′^–GTT ACC TTG TTA CGA CCT–3 ^′^) [32]. Thermal cycling conditions were as follows: an initial denaturation at 94°C for 3 min; 25 cycles of 94°C for 1 min, 50°C for 1 min, and 72°C for 2 min; and a final extension at 72°C for 3 min. Purified PCR products from the GenepHlow Gel/PCR Kit (Geneaid, Taiwan) were sequenced using primers 27F, 518F, 800R, and 1492R. The resulting sequences were assembled (BioEdit) and identified via BLASTn analysis against the GenBank database [33], with pairwise sequence similarity calculated using a global alignment algorithm [34].

### 2.4. Screening of Key Nutritional Components Enhancing Microbial Growth Using PBD

To determine the key nutritional factors influencing the growth of PLA plastic waste–degrading microorganisms, the PBD was employed, based on the methodology described by Liu et al. [35]. Thirteen nutritional components, dissolved in distilled water, were evaluated at a high (+1) and a low (−1) level (Table S1): carbon sources (glucose and sucrose), nitrogen sources (tryptone, beef extract, gelatin, peptone, yeast extract, and (NH_4_)_2_SO_4_), and mineral salts (K_2_HPO_4_, KH_2_PO_4_, MgSO_4_·7H_2_O, ferrous sulfate [FeSO_4_], and sodium chloride [NaCl]).

For each of the 20 experimental runs (Table S2), a 10% (*v*/*v*) cell suspension (OD_600_ = 1.0) was inoculated into 100 mL of the respective PBD medium containing 2 g of sterile PLA coupons. The preparation of the cell suspension and PLA coupons followed the same procedure as described in Section 2.2. Cultures were incubated at 37°C for 24 h with 150 rpm agitation. The initial pH of all PBD media was adjusted to 7.0. After which, microbial growth was assessed by measuring the final OD_600_. The data were analyzed using Stat‐Ease 360 software, and factors with a significance level of *p* < 0.05 were identified as key nutritional components.

### 2.5. Optimization of Nutritional Components for Enhanced PLA Plastic Waste Degradation Using RSM Based on BBD

RSM with a BBD was used to optimize the significant nutritional components identified from the PBD screening [19, 20]. For each isolate, an isolate‐specific BBD matrix was generated using Stat‐Ease 360 software, with each significant factor tested at three levels: low (−1), central (0), and high (+1). Each isolate was optimized independently, with factor level ranges adjusted based on preliminary experiments. The experimental runs were conducted by inoculating each medium (initial pH adjusted to 7.0) and incubating at 37°C for 24 h with 150 rpm agitation. The resulting cell density (OD_600_) was used as the response variable, justified by the assumption that higher biomass leads to greater PLA degradation. Data were subsequently fitted to a quadratic polynomial model, and the effects of variables and their interactions were visualized using three‐dimensional (3D) response surface plots.

To validate the model and confirm the enhancement of PLA plastic waste degradation, each isolate was cultivated in triplicate in 100 mL of its predicted optimal medium (initial pH adjusted to 7.0) containing 2 g of PLA coupons, with shaking at 150 rpm at 37°C. After 24 h, samples were collected, and the OD_600_ was measured to assess the model′s accuracy. The assessment was based on both whether the experimental values fell within the model′s 95% prediction interval (PI) and the percentage error between the predicted and experimental values. Subsequently, the cultivation was continued for a total of 30 days to evaluate the actual PLA degradation. The percentage weight loss of the PLA coupons was then calculated, and surface morphology was analyzed by SEM. Finally, the efficacy of the optimization was confirmed by comparing the percentage weight loss in the optimized medium to that obtained in the initial BM using an independent samples *t*‐test (*p* < 0.05).

## 3. Results and Discussion

### 3.1. Isolation of PLA‐Degrading Microorganisms

PLA‐degrading microorganisms were successfully isolated from samples that were first enriched in situ by a 30‐day PLA burial, a method shown to enhance microbial adaptation and selection in plastic‐contaminated environments [18], before subsequent enrichment cycles in BM broth. Selective enrichment using BM containing only emulsified PLA as the carbon source has been shown to allow the growth of only PLA‐degrading microbes [36]. After enrichment and plating, colonies exhibiting clear zones indicating PLA hydrolysis [19, 37] were observed, with mean diameters of 11.46 ± 0.41 mm (SNRUSAC1), 12.11 ± 0.79 mm (SNRUSAC3), 10.43 ± 0.40 mm (SNRUSAW1), 11.53 ± 0.30 mm (SNRUSAW2), 11.32 ± 0.45 mm (SNRUSAW3), and 11.06 ± 0.20 mm (SNRUSAW4). This suggests the secretion of extracellular enzymes that hydrolyze the ester bonds in PLA [10, 38]. A total of six morphologically distinct isolates (two from compost [SNRUSAC1 and SNRUSAC3] and four from waste dump soil [SNRUSAW1–4]) were selected for further screening of their PLA plastic waste degradation capabilities.

### 3.2. Screening of PLA Plastic Waste–Degrading Microorganisms

All six isolates demonstrated the potential to use postconsumer PLA packaging waste as their sole carbon source, as confirmed by increasing OD_600_ values over time [25]. Having previously shown the ability to degrade pure PLA, the isolates′ growth on this more complex packaging waste confirmed their degradative capacity, a finding consistent with previous reports [18]. The degradation efficiency depends on environmental conditions [39–41], PLA′s physicochemical properties [41, 42], and particularly the microbial strain, including its growth and degradation activity.

However, using OD_600_ to determine a traditional growth curve is not feasible in this system, where PLA serves as the sole carbon source. This recalcitrant substrate limits growth by the slow, rate‐limiting enzymatic release of soluble carbon [43]. Consequently, as OD_600_ measures total turbidity (both viable and dead cells) and not just viable cell counts, the curve is expected to show a prolonged upward trend, making it unsuitable for determining a true stationary phase.

**Figure 1:**
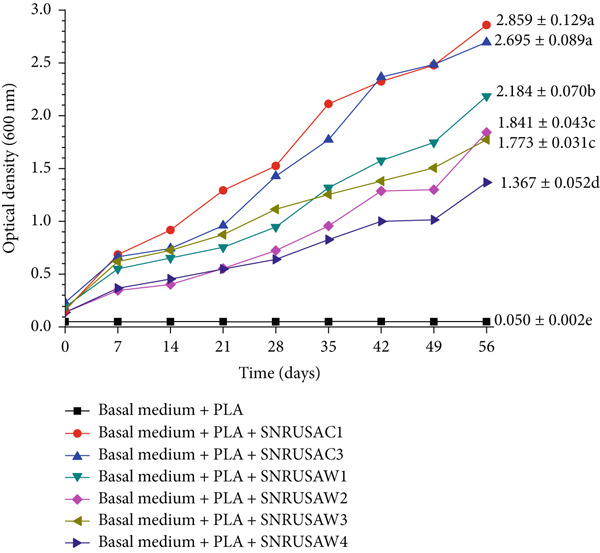
Growth curves of strains SNRUSAC1, SNRUSAC3, SNRUSAW1, SNRUSAW2, SNRUSAW3, and SNRUSAW4 in BM containing PLA plastic waste as the sole carbon source. Microbial growth was monitored by measuring OD_600_ every 7 days for up to 56 days. BM containing PLA plastic waste without inoculation was used as a control. Data at Day 56 are presented as means ± SD (*n* = 3); different letters indicate statistically significant differences as determined by one‐way ANOVA with Tukey′s HSD test (*p* < 0.05).

Despite this limitation, the 56‐day monitoring was sufficient to identify clear performance trends. Over this period, SNRUSAC1 and SNRUSAC3 demonstrated a clearly superior growth trend and ultimately exhibited the highest growth (OD_600_ = 2.859 and 2.695, respectively), which was significantly higher than that of the other isolates (Figure 1). Therefore, all isolates were subsequently assessed for their definitive degradative capabilities via percentage weight loss and SEM analysis.

**Figure 2:**
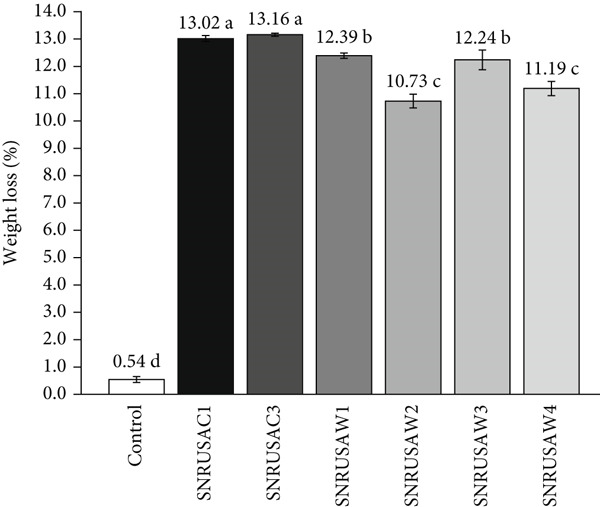
Weight loss percentage of PLA plastic waste coupons after 56 days of incubation with strains SNRUSAC1, SNRUSAC3, SNRUSAW1, SNRUSAW2, SNRUSAW3, and SNRUSAW4 in BM containing PLA plastic waste. BM containing PLA plastic waste without inoculation was used as a control. Bars represent mean values (*n* = 3) with standard deviation; different letters indicate statistically significant differences as determined by one‐way ANOVA with Tukey′s HSD test (*p* < 0.05).

After 56 days of incubation, biodegradation was quantitatively assessed by measuring the percentage weight loss of the PLA coupons, a standard method in polymer degradation studies [19, 20, 44–47]. All inoculated treatments resulted in substantial weight loss (> 10%), significantly exceeding the minimal 0.54% loss observed in the noninoculated control, which was attributed to abiotic hydrolysis. This baseline degradation is a multistep chemical process involving water diffusion into the polymer, followed by the scission of ester bonds in both amorphous and crystalline regions [12]. Zhang et al. [48], who studied abiotic hydrolysis under conditions (37°C, neutral pH buffer, 60 days) that are similar to this study, reported that neat PLA showed almost no mass loss, but a wide range of degradation (from 0.24% to as high as 12.00%) for various PLA composites, depending on the additives. Although the abiotic hydrolysis of this postconsumer PLA (an uncharacterized composite) resulted in a minimal weight loss of only 0.54%, the degradation was substantially accelerated in the presence of microorganisms. Among the isolates, SNRUSAC3 and SNRUSAC1 proved most effective, exhibiting the highest PLA weight loss (13.16% and 13.02%, respectively), which was significantly higher (*p* < 0.05) than that of the other isolates (Figure 2).

The surface morphology of the PLA coupons after 56 days of incubation was examined by SEM (Figure 3). The untreated control surface appeared rough but intact (Figure 3a). In comparison, the noninoculated abiotic control showed small surface pits (Figure 3b), which were likely caused by minor abiotic hydrolysis [48] and consistent with the minimal weight loss previously observed. In contrast, all microbially inoculated coupons showed clear signs of biodegradation. Those treated with strains SNRUSAW1–4 displayed noticeable surface erosion and pore formation (Figures 3e, 3f, 3g, and 3h). Notably, the most extensive damage was observed on coupons incubated with SNRUSAC1 and SNRUSAC3, which featured numerous large, deep pores indicative of widespread matrix breakdown (Figure 3c,d). These surface morphological changes serve as strong qualitative evidence of biodegradation, consistent with the well‐established use of SEM to confirm microbial activity [47, 49, 50], and therefore corroborate the quantitative weight loss data, confirming that SNRUSAC1 and SNRUSAC3 are highly efficient PLA‐degrading strains capable of causing substantial structural damage to the polymer surface.

Figure 3SEM micrographs of PLA plastic waste surfaces: (a) original PLA plastic waste before incubation; (b) PLA plastic waste incubated in BM medium without inoculation; and PLA plastic waste incubated in BM medium with strains (c) SNRUSAC1, (d) SNRUSAC3, (e) SNRUSAW1, (f) SNRUSAW2, (g) SNRUSAW3, and (h) SNRUSAW4 for 56 days. All samples were observed at 5000× magnification.

**Figure:**
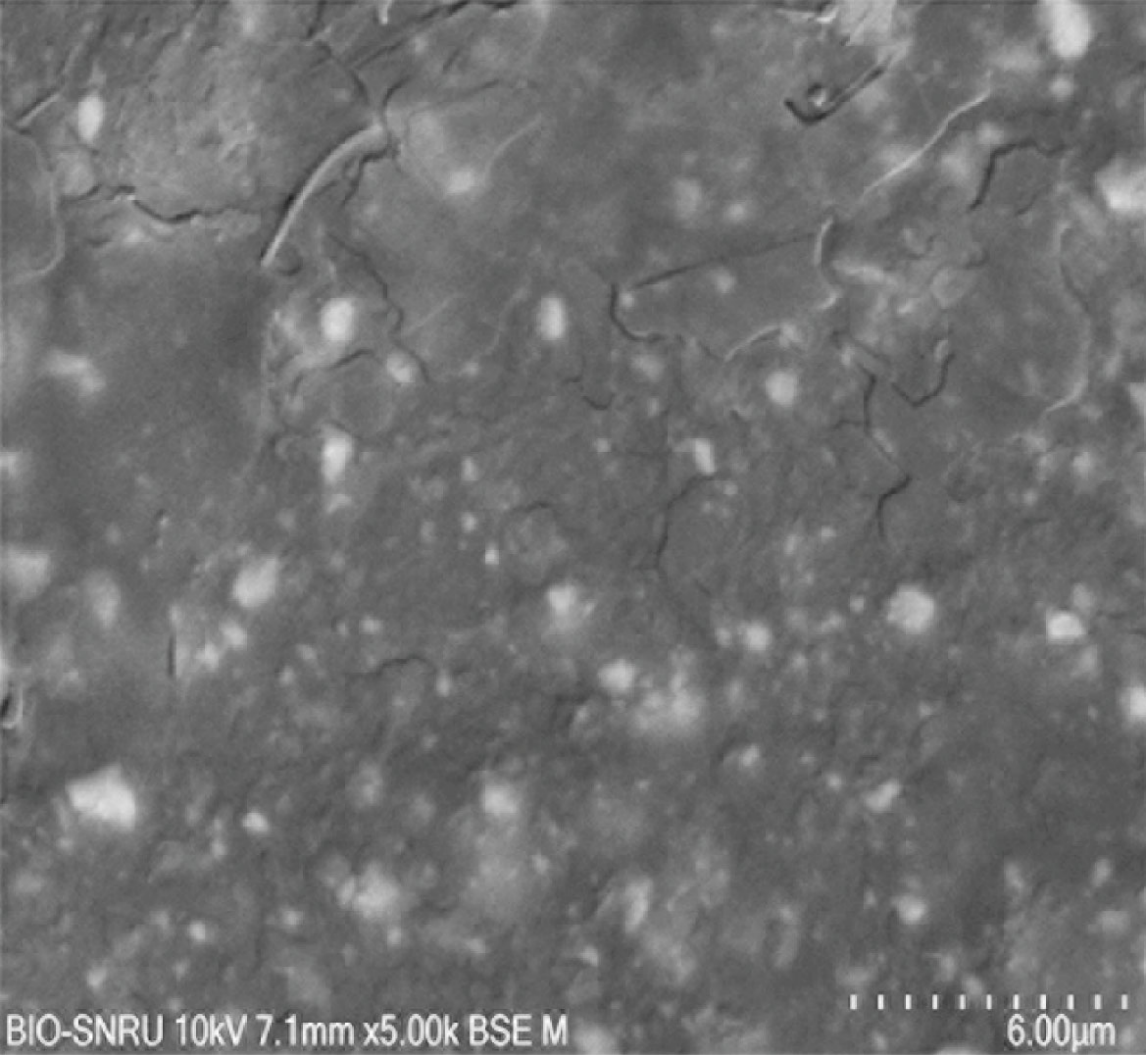
(a)

**Figure:**
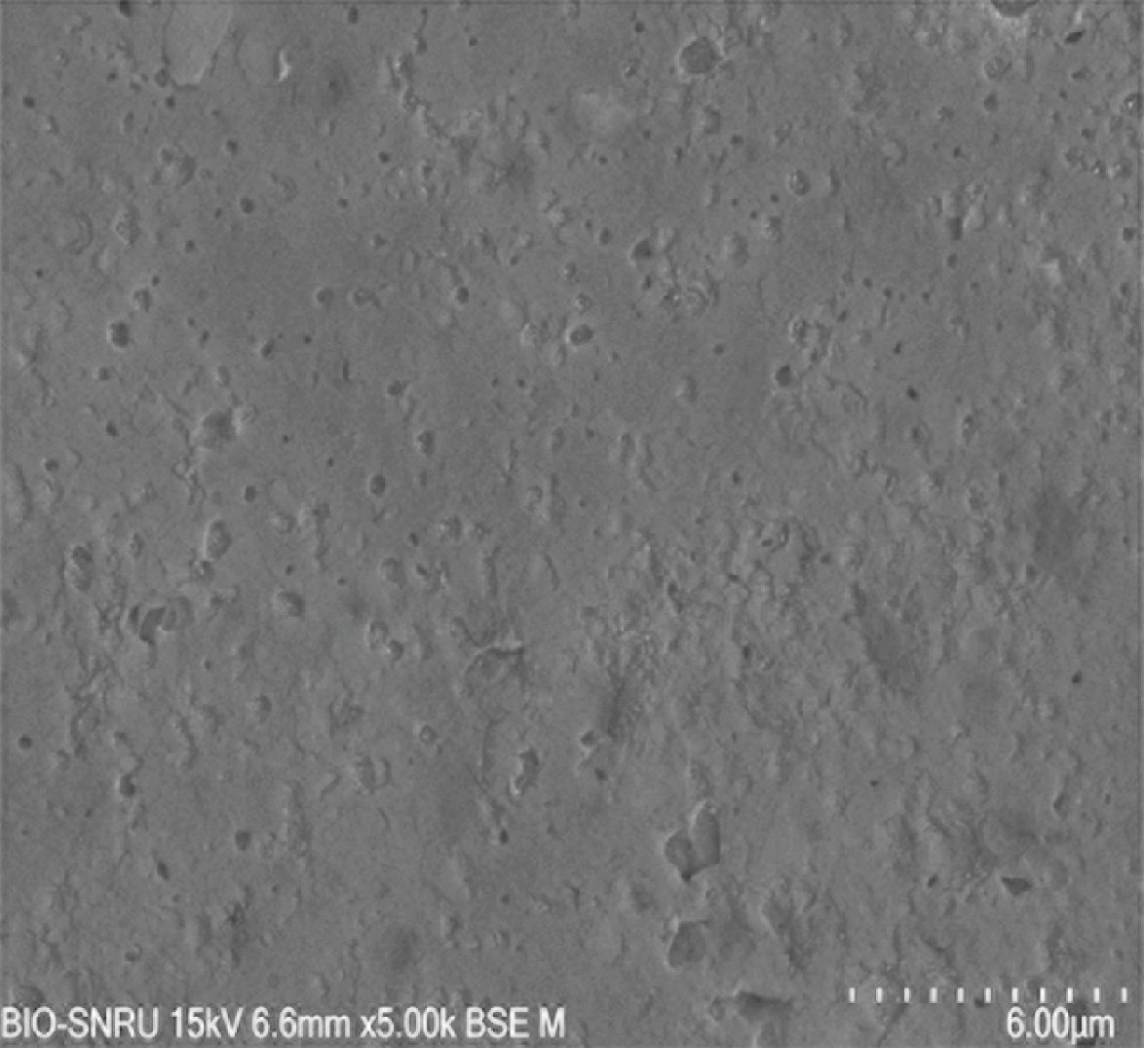
(b)

**Figure:**
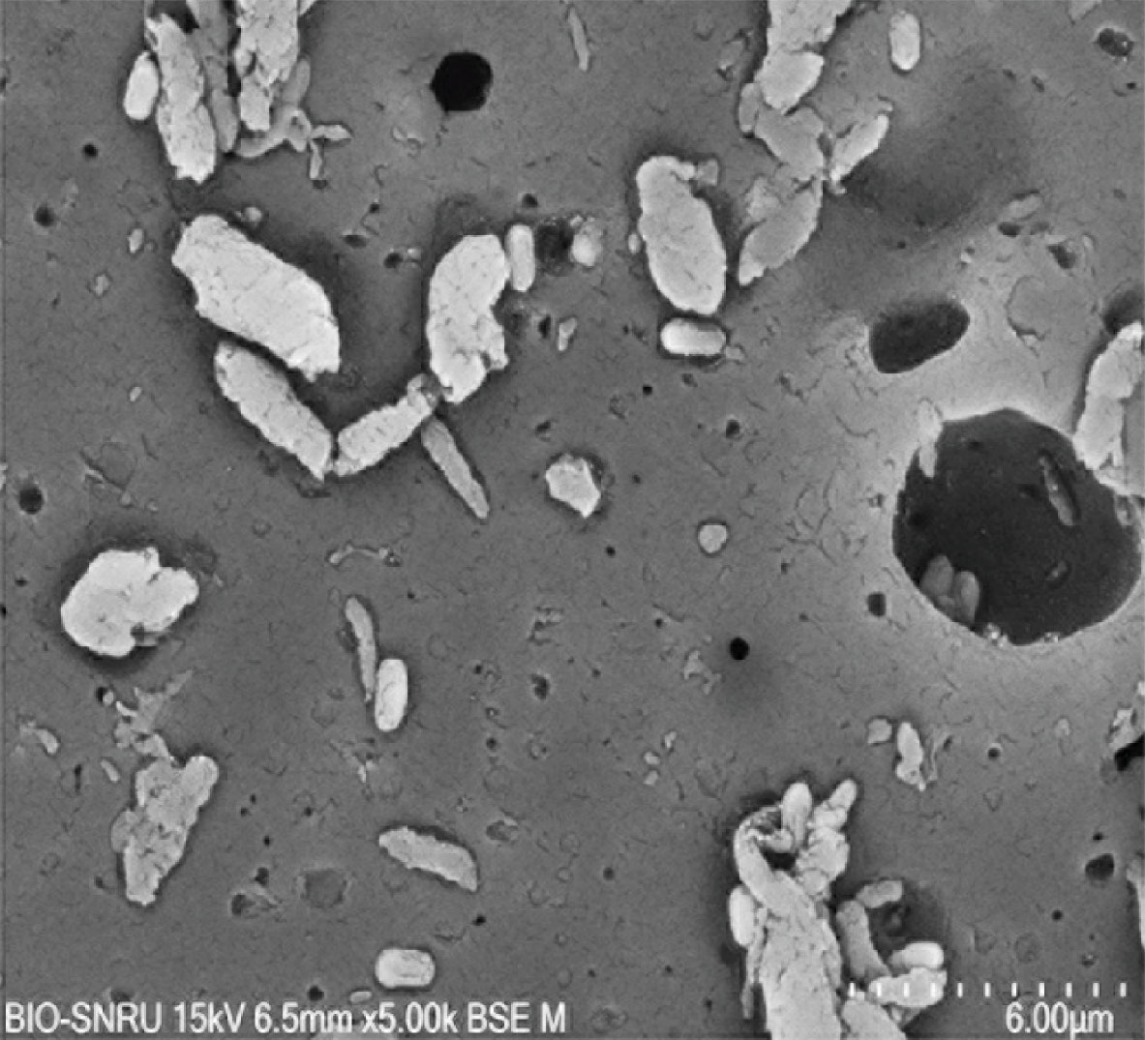
(c)

**Figure:**
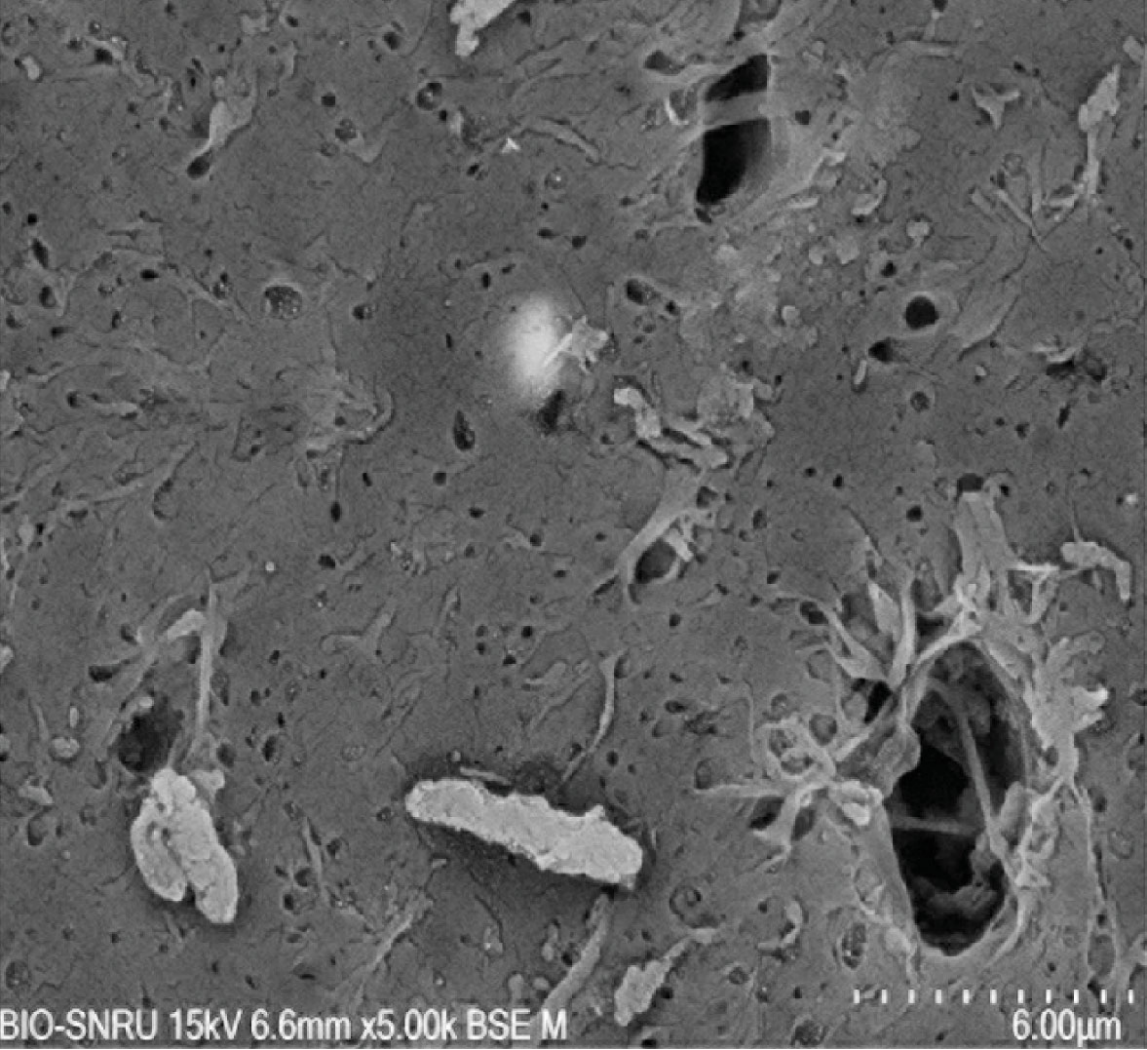
(d)

**Figure:**
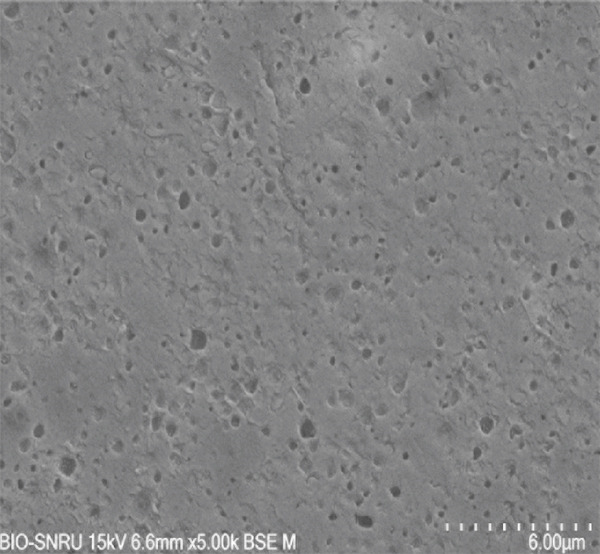
(e)

**Figure:**
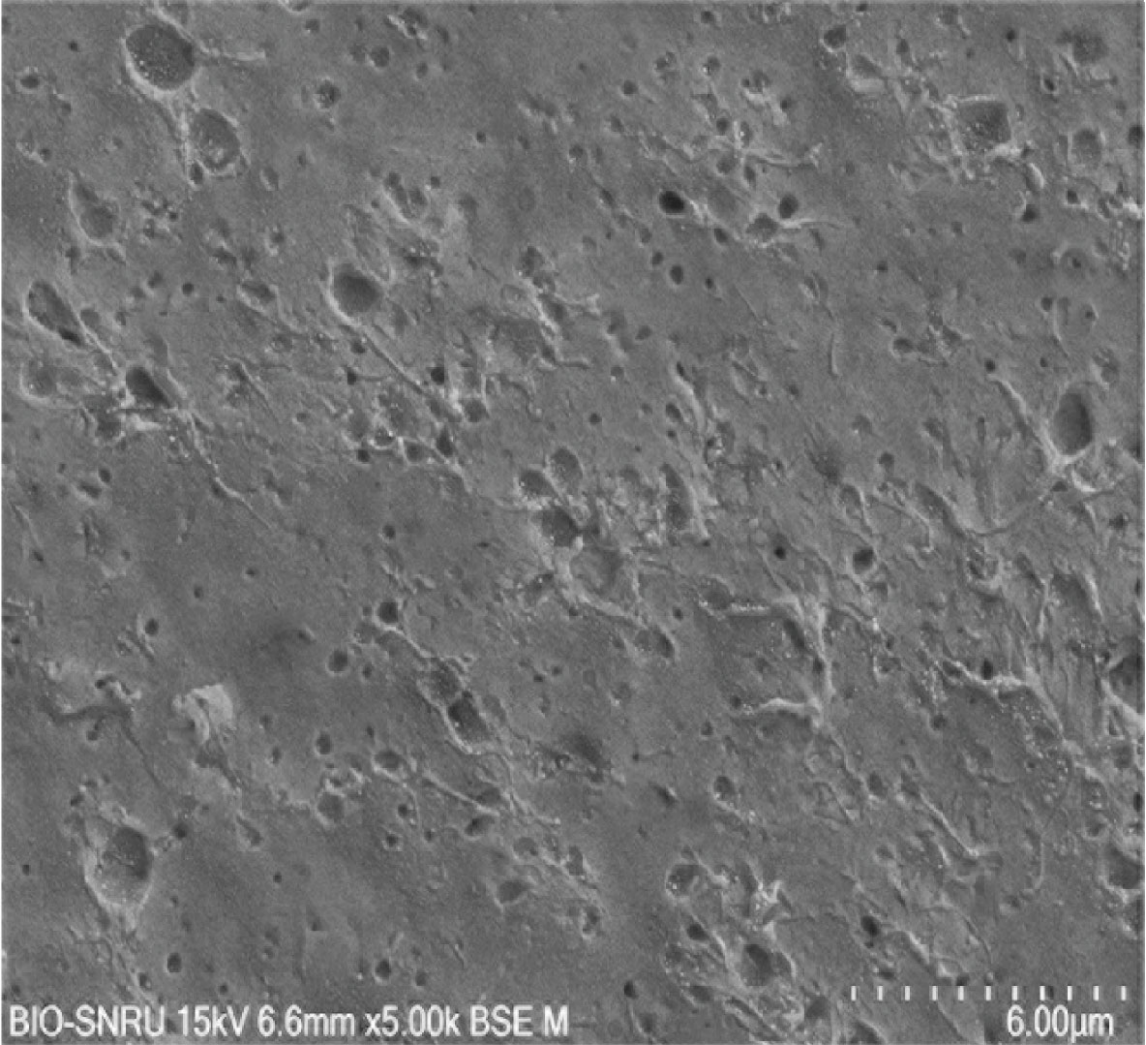
(f)

**Figure:**
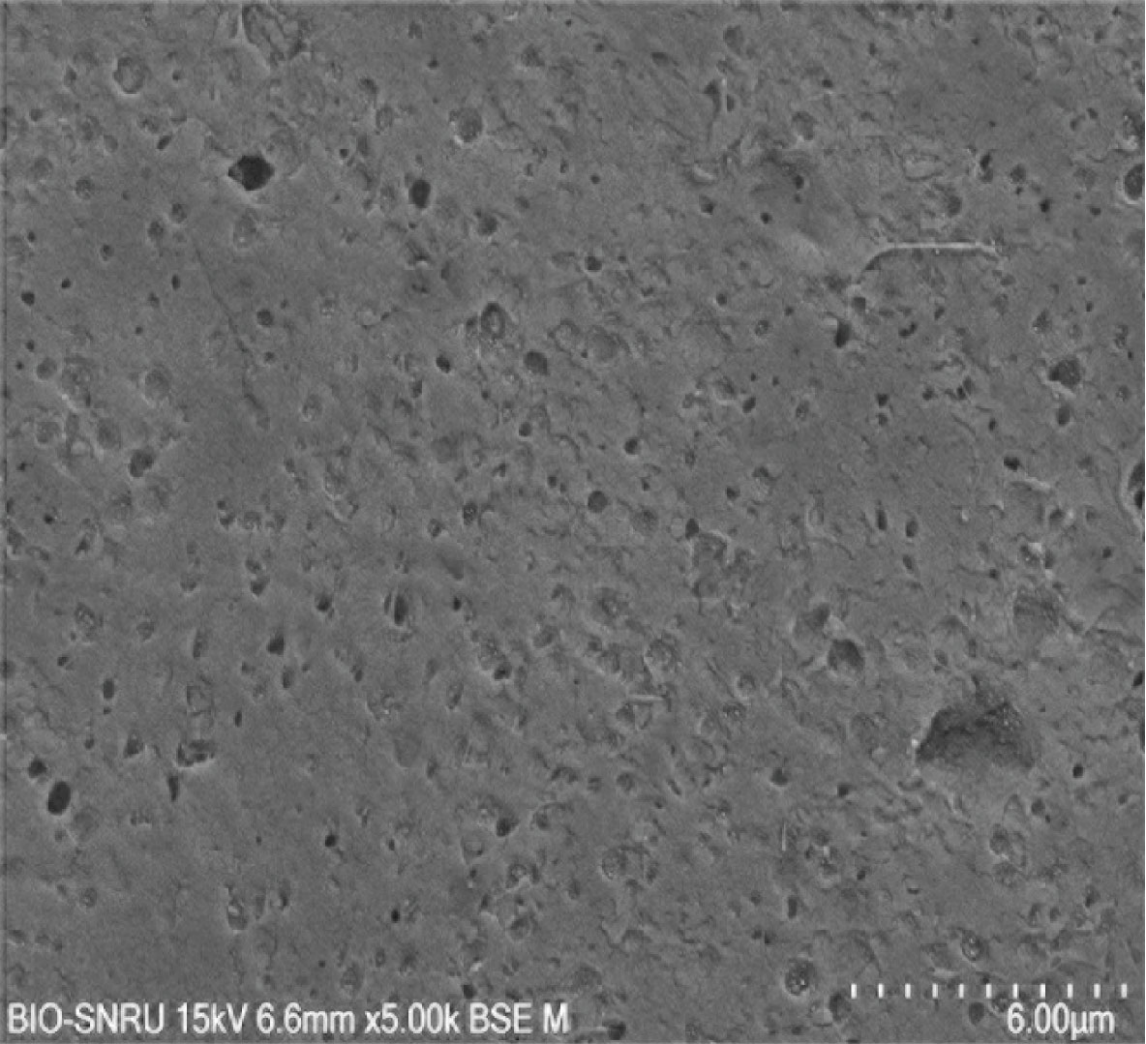
(g)

**Figure:**
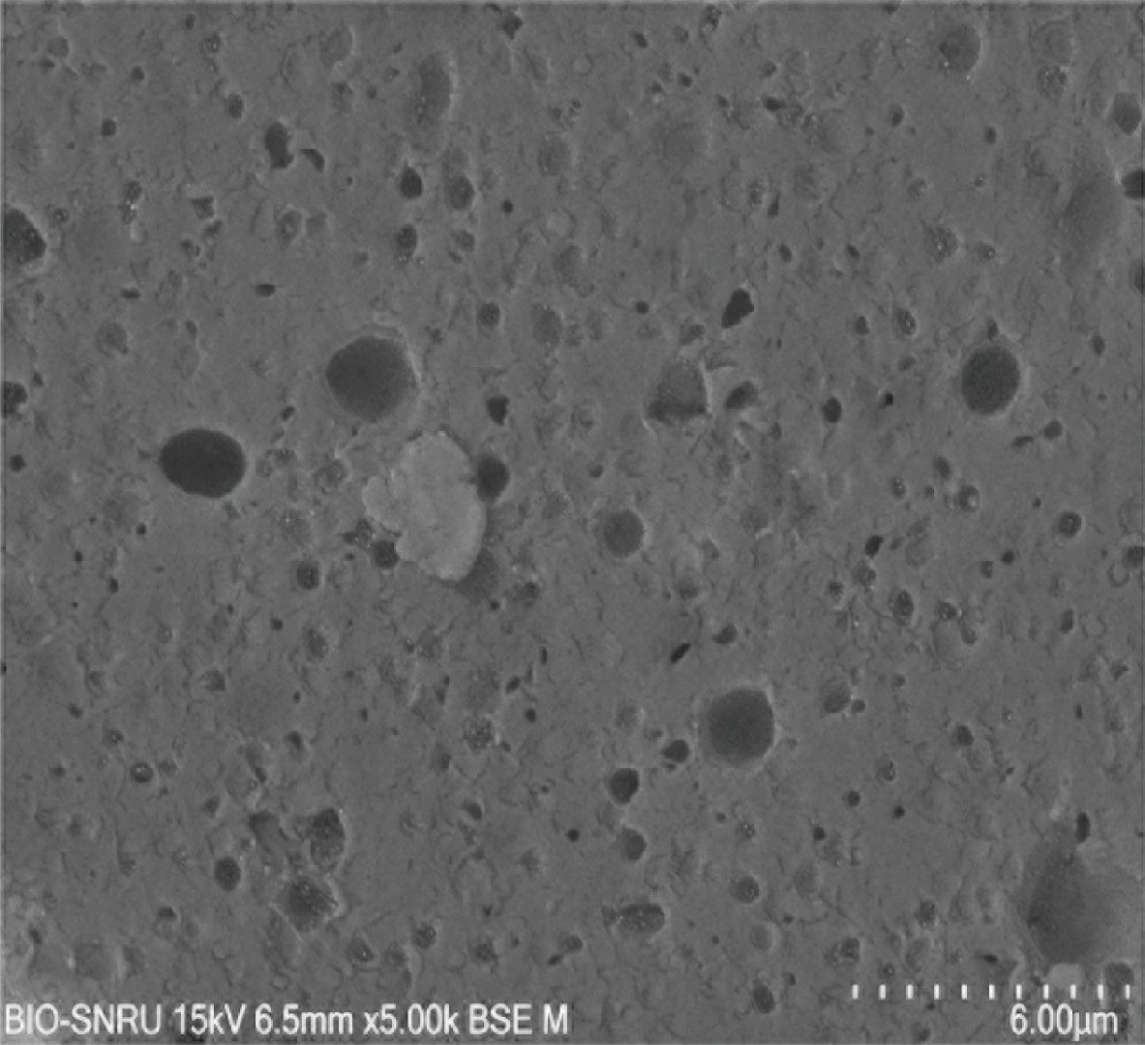
(h)

Based on their superior performance in growth, weight loss, and surface degradation assays, SNRUSAC1 and SNRUSAC3 were identified as the most effective PLA‐degrading strains. Consequently, they were selected for identification and optimization of their degradation conditions using PBD.

To elucidate the potential mechanisms behind this substantial degradation, the two most effective strains, SNRUSAC1 and SNRUSAC3, were assessed for their ability to produce key extracellular enzymes. Qualitative plate assays confirmed that both SNRUSAC1 and SNRUSAC3 produced clear hydrolysis zones for both esterase/lipase (on Tributyrin Agar) and protease (on Skim Milk Agar), indicating their capability to secrete these PLA‐degrading enzymes (Figure S1).

This combined evidence confirms that the degradation was primarily driven by microbial activity. This process involves the secretion of various PLA‐degrading enzymes that hydrolyze PLA′s ester bonds. These enzymes include the lipases/esterases and proteases confirmed in our assays (Figure S1), as well as other depolymerases and cutinases reported in the literature [10, 51, 52]. The resulting lactic acid is then assimilated by the microbes for growth [10], while other secreted organic acids may further accelerate depolymerization [38]. This comprehensive enzymatic action explains both the substantial quantitative weight loss (Figure 2) and the extensive structural disruption (Figure 3) observed. While it is well established that degradation can be significantly accelerated by chemical hydrolysis under alkaline conditions [53], the focus of this study was specifically on the microbial degradation occurring under neutral pH conditions.

Notably, both top‐performing strains originated from compost, a microbial‐rich environment known to harbor polymer‐degrading microorganisms. This finding aligns with previous studies [36, 54], reinforcing compost as a promising source for isolating potent PLA degraders. The adaptation of these strains to the compost environment likely conferred upon them the enhanced enzymatic systems and metabolic plasticity required for efficient PLA degradation. This approach is strongly supported by recent studies, such as Mistry et al. [55], which confirmed that bioaugmentation with a defined consortium is a key strategy for achieving efficient PLA degradation in composting systems. Therefore, their performance under more complex, realistic conditions, such as in commercial composting systems, warrants further investigation. Such studies would also be crucial in evaluating their full potential for future microbial valorization strategies aimed at creating a circular economy for PLA waste.

### 3.3. Identification of Selected Microorganisms

#### 3.3.1. Identification of SNRUSAC1

The morphological and biochemical properties of SNRUSAC1 are presented in Figure S2. On NA, the strain formed circular, creamy‐white, and butyrous colonies with a rough, raised surface and entire margins (Figure S2a). Phase‐contrast microscopy revealed that SNRUSAC1 consists of Gram‐positive, rod‐shaped cells (2–4 *μ*m in length and ~1 *μ*m in width) containing central to subterminal endospores. The cells were observed singly, in pairs, or in small clusters (Figure S2b). These characteristics are consistent with the *Bacillus* genus, a conclusion supported by biochemical tests that identified the isolate as *Bacillus* sp. with 90% probability (Figure S2c).

Analysis of the 16S rDNA sequence, interpreted using the established 98.7% species demarcation threshold [56, 57], revealed that the strain SNRUSAC1 belonged to the genus *Bacillus* but could not be assigned to a specific species. The sequence did not show 100% identity with any known species, though it did show high similarity (≥ 98.7%) to 15 *Bacillus* species. The closest relatives include *Bacillus tequilensis*, *Bacillus cabrialesii*, and *Bacillus inaquosorum* (99.93% similarity; Table S3). The sequence has been deposited in the GenBank database under Accession Number PQ409455.

Based on the combined morphological, biochemical, and molecular characteristics, the strain was designated as *Bacillus* sp. SNRUSAC1. Several *Bacillus* species have been previously reported to degrade PLA, such as *Bacillus brevis* 93 [58], *Bacillus smithii* PL21 [59], *Bacillus stearothermophilus* 73 [60], *Bacillus amyloliquefaciens* MS2 [61], *Bacillus licheniformis* 3 [62], *Bacillus pumilus* B12 [49], *Bacillus* sp. SNRUSA4 [19], and *Bacillus safensis* PLA1006 [63], indicating the relevance of this genus in PLA biodegradation. Members of the *Bacillus* genus are also well known for their high environmental resilience, spore‐forming capability, and adaptability to diverse ecological conditions [64], making them promising candidates for plastic waste biodegradation in natural or engineered environments. The strain′s closest relatives (*B. tequilensis*, *B. cabrialesii*, and *B. inaquosorum*; 99.93% identity) are not known PLA degraders. Furthermore, of the 15 species sharing ≥ 98.7% 16S rDNA identity, only *B. amyloliquefaciens* is a reported degrader. Therefore, these findings identify *Bacillus* sp. SNRUSAC1 as a novel strain with significant potential for biotechnological applications in PLA waste management. Given the limitations of 16S rDNA in resolving closely related *Bacillus* species, whole‐genome sequencing is currently underway to resolve its precise taxonomic status and identify key genes involved in PLA degradation.

#### 3.3.2. Identification of SNRUSAC3

The colony and cell morphology and biochemical properties of strain SNRUSAC3 are examined (Figure S3). On NA, colonies were circular, smooth, glistening, and creamy to pale‐yellowish with a mucoid texture (Figure S3a). Cells were Gram‐positive, rod‐shaped (3–9 *μ*m × 1 *μ*m), occurring singly or in short chains, and formed endospores (Figure S3b). Based on its biochemical reaction pattern (Figure S3c), strain SNRUSAC3 showed a 92% probability of being *Bacillus megaterium*, the basonym of *Priestia megaterium*.

However, molecular analysis revealed that the 1474‐nucleotide 16S rDNA sequence of SNRUSAC3 had 100% identity with *Priestia aryabhattai* B8W22 (GenBank: EF114313) (Table S4). The strain was therefore classified as *P. aryabhattai* SNRUSAC3, and its sequence was deposited under GenBank Accession Number PQ409490.

The discrepancy between the biochemical and molecular results likely stems from the VITEK 2 system′s identification database, which lacks a profile for *B*. *aryabhattai* (now classified as *P*. *aryabhattai* [65]). Consequently, the system defaulted to *P. megaterium* as the most probable match based on available profiles. Notably, *P. megaterium* was also the second‐closest species in the 16S rDNA analysis (99.86% similarity), which underscores the importance of combining molecular tools with biochemical profiling for accurate identification.

Notably, *P. aryabhattai* SNRUSAC3 differed biochemically from the type strain *P. aryabhattai* B8W22 in its reactions to inositol and D‐mannose [66], despite their 100% identical 16S rDNA sequences. These phenotypic differences suggest that *P*. *aryabhattai* SNRUSAC3 is a distinct strain, demonstrating the necessity of integrating multiple approaches to resolve variations within a species.

To date, reports on the degradation of plastics and bioplastics by *P*. *aryabhattai* have included poly(ethylene terephthalate) (PET) [67, 68], acrylonitrile–butadiene–styrene (ABS) [69], and various polyhydroxyalkanoates (PHAs) [70]. These reported PHAs include poly(3‐hydroxybutyrate) (PHB), poly(3‐hydroxybutyrate‐co‐3‐hydroxyhexanoate) (PHBH), and poly(3‐hydroxybutyrate‐co‐3‐hydroxyvalerate) (PHBV) [70]. However, no reports have yet described the degradation of PLA by *P*. *aryabhattai*; therefore, this study represents the first report of PLA degradation by this species. Consequently, *P*. *aryabhattai* SNRUSAC3 can be considered a novel strain capable of PLA degradation.

### 3.4. Identification of Key Nutritional Components Enhancing Microbial Growth

#### 3.4.1. *Bacillus* sp. SNRUSAC1

The effect of 13 nutritional components on the growth of *Bacillus* sp. SNRUSAC1 was investigated using a PBD, with the experimental design and results (OD_600_) presented in Table S2. The OD_600_ values varied significantly across the 20 experimental runs. The highest (OD_600_ = 6.965) and lowest (OD_600_ = 1.312) cell growth were observed in Run Numbers 4 and 19, respectively. ANOVA confirmed the model′s significance for microbial growth (*p* = 0.0463). Among the 13 components tested, yeast extract, tryptone, NaCl, and sucrose were identified as the most significant factors positively influencing the growth of *Bacillus* sp. SNRUSAC1, with *p* values of 0.0043, 0.0192, 0.0198, and 0.0466, respectively (Table 1). The identified key factors of *Bacillus* sp. SNRUSAC1 represent all three tested nutritional categories: a carbon source (sucrose), nitrogen sources (yeast extract and tryptone), and a mineral salt (NaCl), highlighting the importance of each group for promoting growth.

**Table 1:** ANOVA of the PBD for the growth of *Bacillus* sp. SNRUSAC1.

**Term**	**Sum of squares**	**df**	**Mean square**	**F** **-value**	**p** **value**
Model	32.85	13	2.53	4.11	0.0463 ^∗^
*X*_1_ K_2_HPO_4_	0.0552	1	0.0552	0.0899	0.7745
*X*_2_ KH_2_PO_4_	0.1769	1	0.1769	0.2879	0.6109
*X*_3_ MgSO_4_·7H_2_O	0.0456	1	0.0456	0.0742	0.7944
*X*_4_ (NH_4_)_2_SO_4_	1.16	1	1.16	1.88	0.2190
*X*_5_ glucose	1.71	1	1.71	2.78	0.1466
*X*_6_ sucrose	3.84	1	3.84	6.25	0.0466 ^∗^
*X*_7_ tryptone	6.19	1	6.19	10.07	0.0192 ^∗^
*X*_8_ beef extract	1.12	1	1.12	1.83	0.2254
*X*_9_ gelatin	0.1629	1	0.1629	0.2651	0.6251
*X*_10_ peptone	0.0228	1	0.0228	0.0372	0.8535
*X*_11_ yeast extract	12.22	1	12.22	19.89	0.0043 ^∗^
*X*_12_ FeSO_4_	0.0505	1	0.0505	0.0822	0.7840
*X*_13_ NaCl	6.10	1	6.10	9.92	0.0198 ^∗^
Residual	3.69	6	0.6145		
Cor total	36.53	19			

^∗^Statistically significant difference (*p* < 0.05).

The findings for sucrose, tryptone, yeast extract, and NaCl are well supported by the literature. Sucrose is a well‐established effective carbon source for *Bacillus* species, fundamental for cell synthesis and energy production [71]. For nitrogen sources, both tryptone and yeast extract were significant. Tryptone provides key precursors for cell synthesis. For instance, its high content of amino acids such as aspartic acid, glutamine, and glycine serves as a nitrogen donor for synthesizing nucleotides and nucleic acids [72]. Consistent with our findings, media containing tryptone have also been shown to enhance the growth of *Bacillus* species [73]. Similarly, yeast extract, a rich source of amino acids and vitamins, is a well‐established supplement that promotes robust microbial growth [74], including in *Bacillus* [75]. Finally, NaCl is crucial for maintaining cellular osmotic balance during active incubation [76].

#### 3.4.2. *P. aryabhattai* SNRUSAC3

In parallel, the same set of 20 experimental conditions was used to assess the nutritional requirements for the growth of *P. aryabhattai* SNRUSAC3. The corresponding OD_600_ results are detailed in Table S2. *P. aryabhattai* SNRUSAC3 exhibited its maximum growth (OD_600_ = 17.612) in Run Number 9, while its minimum growth (OD_600_ = 5.559) occurred in Run Number 19. Notably, Run 19, the control condition with all 13 variables at their low levels, yielded the lowest growth for both strains. This finding strongly suggests that the basal condition was highly nutrient‐limited and highlights the collective importance of the tested components for the growth of these isolates. The statistical analysis also yielded a significant model (*p* = 0.0265). The screening pinpointed glucose, FeSO_4_, yeast extract, and (NH_4_)_2_SO_4_ as the key components positively affecting growth, with *p* values of 0.0035, 0.0041, 0.0256, and 0.0311, respectively (Table 2). As with *Bacillus* sp. SNRUSAC1, the significant nutritional factors for *P. aryabhattai* SNRUSAC3 were also distributed across all three fundamental categories.

**Table 2:** ANOVA of the PBD for the growth of *P*. *aryabhattai* SNRUSAC3.

**Term**	**Sum of squares**	**df**	**Mean square**	**F** **-value**	**p** **value**
Model	192.36	13	14.80	5.20	0.0265 ^∗^
*X*_1_ K_2_HPO_4_	0.1763	1	0.1763	0.0620	0.8117
*X*_2_ KH_2_PO_4_	1.44	1	1.44	0.5077	0.5029
*X*_3_ MgSO_4_·7H_2_O	0.1728	1	0.1728	0.0608	0.8135
*X*_4_ (NH_4_)_2_SO_4_	22.34	1	22.34	7.85	0.0311 ^∗^
*X*_5_ glucose	61.87	1	61.87	21.75	0.0035 ^∗^
*X*_6_ sucrose	6.23	1	6.23	2.19	0.1893
*X*_7_ tryptone	7.49	1	7.49	2.63	0.1558
*X*_8_ beef extract	5.99	1	5.99	2.10	0.1970
*X*_9_ gelatin	0.1222	1	0.1222	0.0430	0.8427
*X*_10_ peptone	1.61	1	1.61	0.5651	0.4806
*X*_11_ yeast extract	24.79	1	24.79	8.71	0.0256 ^∗^
*X*_12_ FeSO_4_	57.59	1	57.59	20.25	0.0041 ^∗^
*X*_13_ NaCl	2.54	1	2.54	0.8940	0.3809
Residual	17.07	6	2.84		
Cor total	209.43	19			

^∗^Statistically significant difference (*p* < 0.05).

The significant impact of these four components is supported by their well‐documented roles in microbial metabolism. The importance of glucose and yeast extract for *P. aryabhattai* growth has been reported by Korcan et al. [77] and Ojha et al. [27], respectively. (NH_4_)_2_SO_4_ serves as another critical nitrogen source, providing ammonium ions for the synthesis of glutamate and glutamine. These amino acids are essential precursors for producing other vital biomolecules, including amino acids, nucleosides, purines, and pyrimidines [78, 79]. FeSO_4_ provides iron, a crucial element for key metabolic processes, DNA replication, transcription, and energy generation in bacteria [80]. The finding that FeSO_4_ was a significant factor for the growth of *P. aryabhattai* SNRUSAC3 in this study is particularly noteworthy. It aligns with the work of Hamid et al. [81], who successfully isolated *P. aryabhattai*, a strain which they noted for its heavy metal resistance, from soil using a medium containing FeSO_4_, further confirming its role as a growth‐promoting factor for this species. This iron‐scavenging capability provides a significant competitive advantage in nutrient‐rich but highly competitive environments like compost, which explains its successful isolation from such a source.

### 3.5. Enhanced Degradation of PLA Plastic Waste Following Nutritional Optimization

#### 3.5.1. *Bacillus* sp. SNRUSAC1

Based on the preceding screening results, the four most significant components affecting the growth of *Bacillus* sp. SNRUSAC1 were selected for further optimization: yeast extract (*X*_1_), tryptone (*X*_2_), NaCl (*X*_3_), and sucrose (*X*_4_). A BBD was employed within an RSM framework, generating 27 experimental runs. The complete design matrix, along with the observed and model‐predicted responses (OD_600_), is presented in Table S5.

The experimental data were fitted to a second‐order polynomial equation, which includes linear, two‐factor interaction, and quadratic terms. The final predictive equation in terms of actual factors is shown in Equation (1):

(1) Y=−1.1518322114583+0.78538364583333X1+0.39411376666667X2+0.22291223958333X3+0.33972864583333X4−0.0010312499999999X1X2−0.0071354166666667X1X3−0.0019401041666667 X1X4−0.0024375X2X3−0.01446875X2X4+0.00298828125X3X4−0.026098645833333X12−0.0179383X22−0.01982546875X32−0.020167265625X42,

where *Y* is the predicted response (OD_600_) and *X*_1_, *X*_2_, *X*_3_, and *X*_4_ are the actual values of yeast extract, tryptone, NaCl, and sucrose, respectively.

ANOVA was used to evaluate the predictive model′s significance and adequacy (Table 3). The quadratic model was highly significant, with a very low *p* value (< 0.0001) and a high *F*‐value of 65.99. The model′s excellent fit to the experimental data was confirmed by a high coefficient of determination (*R* ^2^ = 0.9872) and an adjusted *R* ^2^ of 0.9722. Furthermore, the predicted *R* ^2^ of 0.9303 was in reasonable agreement with the adjusted *R* ^2^, with a difference of less than 0.2. The adequate precision was 26.3895, well above the desirable threshold of 4. A nonsignificant lack of fit (*p* = 0.3875) further supported the model′s validity. Collectively, these statistical metrics confirm that the model is robust, reliable, and suitable for predicting the growth of *Bacillus* sp. SNRUSAC1. The linear effects of yeast extract (*X*_1_), tryptone (*X*_2_), and sucrose (*X*_4_), as well as all four quadratic terms ($X_{1}^{2}$, $X_{2}^{2}$, $X_{3}^{2}$, and $X_{4}^{2}$), were highly significant (*p* < 0.05). Although the linear effect of NaCl (*X*_3_) itself was not significant (*p* = 0.2227), its significant quadratic effect ($X_{3}^{2}$; *p* = 0.0185) confirms its influence. This indicates that each of the four components significantly influenced the growth of *Bacillus* sp. SNRUSAC1. Conversely, no significant two‐factor interactions were observed (*p* > 0.05), indicating that these components act independently, without any significant synergistic or antagonistic effects among them within the tested range.

**Table 3:** ANOVA for the BBD‐based quadratic model for *Bacillus* sp. SNRUSAC1 growth.

**Source**	**SS**	**df**	**MS**	**F** **-value**	**p** **value**
Model	66.88	14	4.78	65.99	< 0.0001 ^∗^
Yeast extract	58.64	1	58.64	810.05	< 0.0001 ^∗^
Tryptone	2.28	1	2.28	31.44	0.0001 ^∗^
NaCl	0.1198	1	0.1198	1.65	0.2227
Sucrose	0.5313	1	0.5313	7.34	0.0190 ^∗^
Yeast extract∗tryptone	0.0038	1	0.0038	0.0529	0.8220
Yeast extract∗NaCl	0.1173	1	0.1173	1.62	0.2271
Yeast extract∗sucrose	0.0087	1	0.0087	0.1198	0.7353
Tryptone∗NaCl	0.0095	1	0.0095	0.1313	0.7234
Tryptone∗sucrose	0.3350	1	0.3350	4.63	0.0525
NaCl∗sucrose	0.0091	1	0.0091	0.1263	0.7285
Yeast extract∗yeast extract	4.71	1	4.71	65.03	< 0.0001 ^∗^
Tryptone∗tryptone	1.07	1	1.07	14.82	0.0023 ^∗^
NaCl∗NaCl	0.5366	1	0.5366	7.41	0.0185 ^∗^
Sucrose∗sucrose	0.5553	1	0.5553	7.67	0.0170 ^∗^
Residual	0.8687	12	0.0724		
Lack of fit	0.7876	10	0.0788	1.94	0.3875
Pure error	0.0811	2	0.0406		
Cor total	67.75	26			
*R* ^2^ = 0.9872 Adjusted *R* ^2^ = 0.9722 Predicted *R* ^2^ = 0.9303 Adeq precision = 26.3895					

^∗^Statistically significant difference (*p* < 0.05).

To visualize the effects of the independent variables and their interactions on the growth of *Bacillus* sp. SNRUSAC1, 3D response surface and corresponding contour plots were generated from the predictive model (Equation 1). Each plot illustrates the effect of two variables, while the other two were held constant at their central level (Figure 4). An increase in yeast extract concentration from 1 to 13 g/L consistently resulted in enhanced growth (Figures 4a, 4b, and 4c). The optimal concentration for tryptone was observed to be within the range of 7–10 g/L (Figures 4a, 4d, and 4e). The suitable concentration range for NaCl was approximately 2–4 g/L (Figures 4b, 4d, and 4f), while that for sucrose was around 4–6 g/L (Figures 4c, 4e, and 4f). Based on the model′s prediction, the optimal medium composition was determined to be yeast extract, 12.98 g/L; tryptone, 8.76 g/L; NaCl, 3.18 g/L; and sucrose, 5.12 g/L. Under these conditions, a maximum OD_600_ of 7.231 was predicted.

Figure 43D response surface and corresponding contour plots showing the interaction effects of medium components on the growth (OD_600_) of *Bacillus* sp. SNRUSAC1. The interaction between (a) yeast extract and tryptone, (b) yeast extract and NaCl, (c) yeast extract and sucrose, (d) tryptone and NaCl, (e) tryptone and sucrose, and (f) NaCl and sucrose.

**Figure:**
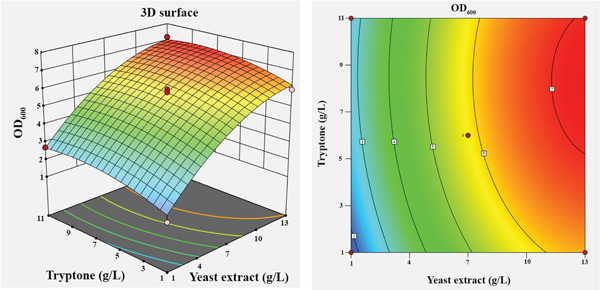
(a)

**Figure:**
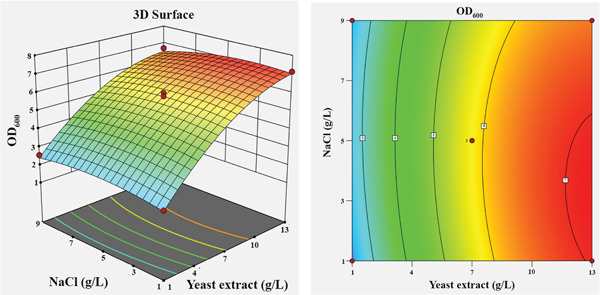
(b)

**Figure:**
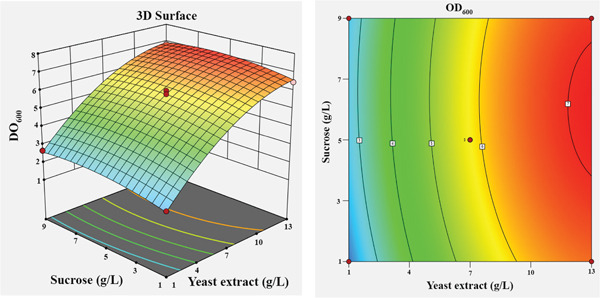
(c)

**Figure:**
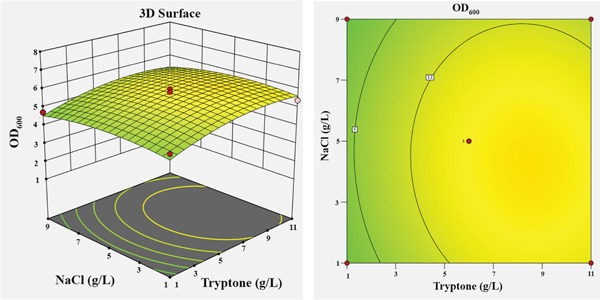
(d)

**Figure:**
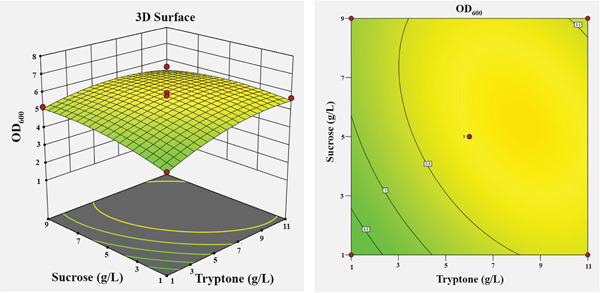
(e)

**Figure:**
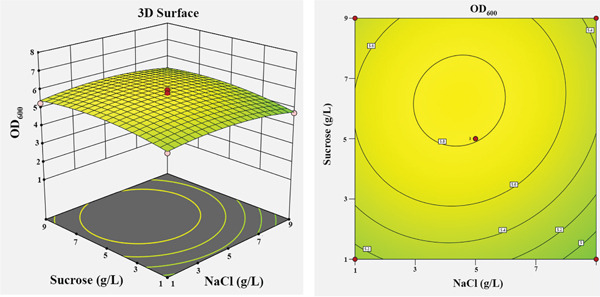
(f)

Yeast extract provides more than just a nitrogen source; its richness in sulfur, B‐vitamins, and essential growth factors is vital for promoting *Bacillus* growth [82] and enhancing PLA degradation by *Bacillus* [19]. Regarding the complex nitrogen sources, the individual significance of both yeast extract and tryptone highlights their complementary roles in achieving optimal performance for *Bacillus* sp. SNRUSAC1. This observation aligns with Pan et al. [83], who demonstrated the benefit of utilizing these two components in combination for *B. subtilis* D21‐8 cultivation. Furthermore, the optimal tryptone concentration reported in their study (7.9 g/L) falls squarely within our study′s optimal range (7–10 g/L), suggesting a comparable metabolic requirement among these *Bacillus* strains.

The significance of NaCl is broadly linked to its role in maintaining cellular osmotic balance, a critical factor for bacterial survival in varying environments [76]. However, the optimal NaCl concentration is highly strain‐specific and necessitates individual evaluation, a point clearly illustrated by the diversity observed among *Bacillus* isolates. For instance, the optimal range for *Bacillus* sp. SNRUSAC1 in our study was found to be relatively low at 2–4 g/L (0.2%–0.4%). This contrasts sharply with strains adapted to saline environments, such as the marine isolate *B*. *subtilis* D21‐8, which was cultivated in a medium containing as high as 30 g/L (3%) NaCl [83]. This wide spectrum of adaptation is further highlighted by reports of strains with even more extreme characteristics; some possess extreme halotolerance, surviving in up to 20% NaCl [84], while others, like *B. altitudinis* WR10, require no NaCl for optimal growth yet can tolerate concentrations as high as 12% [85]. This diversity underscores the importance of empirical optimization for each specific isolate. Although our findings pinpoint an optimal growth range for *Bacillus* sp. SNRUSAC1, its maximum halotolerance remains uninvestigated and represents a promising avenue for future research into its broader environmental applications.

The optimal sucrose concentration range in our study was 4–6 g/L. This finding is consistent with existing literature, which reports that sucrose is an effective carbon source for *Bacillus* species. However, the optimal concentration can vary significantly between different isolates, reflecting their unique metabolic requirements. For instance, while *Bacillus* sp. SNRUSAC1 thrives at a relatively low sucrose concentration, this observation aligns well with findings for other isolates when their growth data are examined closely. In the work of Harun et al. [86], although the peak biomass for their *Bacillus* sp. isolate was recorded at 10 g/L, it is noteworthy that substantial, near‐maximal growth was already achieved at 5 g/L. This latter observation is particularly consistent with the 4–6 g/L optimal range identified in our study.

To validate the model′s predictive accuracy, a confirmation experiment was conducted using the optimized medium, which contained 12.98 g/L of yeast extract, 8.76 g/L of tryptone, 3.18 g/L of NaCl, and 5.12 g/L of sucrose. After 24 h of cultivation with PLA coupons, the mean experimental OD_600_ was 7.274 ± 0.163. This observed value falls comfortably within the 95% PI of 6.54–7.92 generated by the model. Furthermore, the percentage error between the predicted (7.231) and the observed mean was only 0.59%. These results collectively demonstrate the high accuracy, validity, and reliability of the developed RSM model.

**Figure 5:**
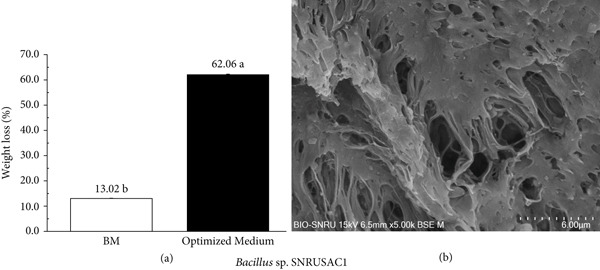
Enhanced degradation of PLA plastic waste by *Bacillus* sp. SNRUSAC1 in the optimized medium. (a) Comparison of PLA coupon percentage weight loss after incubation in the initial BM for 56 days and the optimized medium for 30 days. Data are presented as means ± SD (*n* = 3); different letters indicate a statistically significant difference as determined by an independent samples *t*‐test (*p* < 0.05). (b) SEM images showing the surface morphology of the PLA coupon after 30 days of degradation in the optimized medium (magnification, 5000×).

Crucially, the optimization of the medium led to a dramatic enhancement in PLA plastic waste degradation. The percentage weight loss of the PLA coupons increased significantly from 13.02*%* ± 0.11*%* after 56 days in the BM to 62.06*%* ± 0.21*%* in only 30 days under the optimized medium (Figure 5a). This degradation efficiency is notably high; for comparison, a recent study by Wang et al. [63], investigating a different *Bacillus* strain (*B. safensis*) under similar mesophilic conditions, reported only ~8% weight loss over the same 30‐day period. This enhanced degradation was corroborated by SEM analysis (Figure 5b), which revealed extensive erosion and pronounced disintegration of the PLA surface, resulting in a highly porous morphology with numerous large cavities penetrating deep into the inner layers of the material. This clearly demonstrates a strong positive correlation between the enhanced growth of *Bacillus* sp. SNRUSAC1 and its PLA degradation efficiency.

#### 3.5.2. *P. aryabhattai* SNRUSAC3

To enhance the PLA degradation potential of *P*. *aryabhattai* SNRUSAC3, an optimization of its growth medium was conducted using RSM. Based on the initial screening, glucose, yeast extract, (NH_4_)_2_SO_4_, and FeSO_4_ were identified as the four components with the most significant statistical influence on cell growth (OD_600_) and were therefore selected as the independent variables. A BBD was utilized to generate a 27‐run experimental matrix. The complete design, detailing both the experimentally observed and model‐predicted responses, is presented in Table S6.

A second‐order polynomial equation was generated from the experimental data to model the effects of the four variables, incorporating linear, two‐factor interaction, and quadratic terms. The resulting predictive model in terms of actual factors is presented as Equation (2):

(2) Y=0.19928309375003+0.86971744791666X1+1.8436793333333X2−1.1402630208333X3+14.778260416667X4+0.035765625X1X2−0.0369140625X1X3−0.77109375X1X4+0.00065625000000012X2X3+0.6015625X2X4−1.1046875X3X4−0.017772916666666X12−0.11414341666667X22+0.22312708333333X32−1.1755729166666X42.

In this equation, *Y* represents the predicted growth (OD_600_), while *X*_1_, *X*_2_, *X*_3_, and *X*_4_ are the actual concentrations of glucose, yeast extract, (NH_4_)_2_SO_4_, and FeSO_4_, respectively.

ANOVA was performed to assess the statistical significance and validity of the model (Table 4). A very low *p* value (< 0.0001) and an *F*‐value of 39.41 demonstrated that the quadratic model was highly significant. The model′s excellent fit was substantiated by a high coefficient of determination (*R* ^2^ = 0.9787) and an adjusted *R* ^2^ of 0.9539. The predicted *R* ^2^ of 0.8798 showed good concordance with the adjusted *R* ^2^, and a nonsignificant lack of fit (*p* = 0.1490) further confirmed the model′s reliability. Moreover, the adequate precision of 20.8061 indicated an excellent signal‐to‐noise ratio. These results confirm that the model is suitable for accurately predicting the growth of *P*. *aryabhattai* SNRUSAC3. In terms of individual factor effects, the linear terms for glucose (*X*_1_), yeast extract (*X*_2_), (NH_4_)_2_SO_4_ (*X*_3_), and FeSO_4_ (*X*_4_) were all highly significant (*p* < 0.0001, < 0.0001, 0.0011, and < 0.0001, respectively). Conversely, no significant two‐factor interactions were observed (*p* > 0.05). Among the quadratic terms, both yeast extract ($X_{2}^{2}$) and (NH_4_)_2_SO_4_ ($X_{3}^{2}$) had a significant effect on growth (*p* < 0.0001 and *p* = 0.0356, respectively).

**Table 4:** ANOVA for the BBD‐based quadratic model for *P*. *aryabhattai* SNRUSAC3 growth.

**Source**	**SS**	**df**	**MS**	**F** **-value**	**p** **value**
Model	418.28	14	29.88	39.41	< 0.0001 ^∗^
Glucose	65.25	1	65.25	86.07	< 0.0001 ^∗^
Yeast extract	209.12	1	209.12	275.84	< 0.0001 ^∗^
(NH_4_)_2_SO_4_	13.82	1	13.82	18.24	0.0011 ^∗^
FeSO_4_	58.77	1	58.77	77.52	< 0.0001 ^∗^
Glucose∗yeast extract	2.05	1	2.05	2.70	0.1263
$\text{Glucose}*{\left(\text{N}{\text{H}}_{4}\right)}_{2}\text{S}{\text{O}}_{4}$	0.3488	1	0.3488	0.4601	0.5104
Glucose∗FeSO_4_	1.52	1	1.52	2.01	0.1819
$\text{Yeast}\;\text{extract}*{\left(\text{N}{\text{H}}_{4}\right)}_{2}\text{S}{\text{O}}_{4}$	0.0002	1	0.0002	0.0002	0.9882
Yeast extract∗FeSO_4_	1.45	1	1.45	1.91	0.1922
${\left(\text{N}{\text{H}}_{4}\right)}_{2}\text{S}{\text{O}}_{4}*\text{FeS}{\text{O}}_{4}$	0.7810	1	0.7810	1.03	0.3301
Glucose∗glucose	0.4313	1	0.4313	0.5689	0.4652
Yeast extract∗yeast extract	43.43	1	43.43	57.29	< 0.0001 ^∗^
${\left(\text{N}{\text{H}}_{4}\right)}_{2}\text{S}{\text{O}}_{4}*{\left(\text{N}{\text{H}}_{4}\right)}_{2}\text{S}{\text{O}}_{4}$	4.25	1	4.25	5.60	0.0356 ^∗^
FeSO_4_∗FeSO_4_	0.0118	1	0.0118	0.0156	0.9028
Residual	9.10	12	0.7581		
Lack of fit	8.81	10	0.8809	6.10	0.1490
Pure error	0.2888	2	0.1444		
Cor total	427.38	26			
*R* ^2^ = 0.9787 Adjusted *R* ^2^ = 0.9539 Predicted *R* ^2^ = 0.8798 Adeq precision = 20.8061					

^∗^Statistically significant difference (*p* < 0.05).

**Figure 7:**
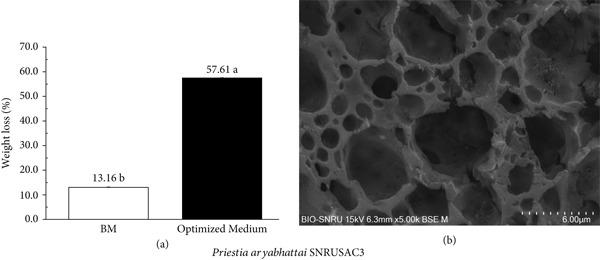
Enhanced degradation of PLA plastic waste by *P. aryabhattai* SNRUSAC3 in the optimized medium. (a) Comparison of PLA coupon percentage weight loss after incubation in the initial BM for 56 days and the optimized medium for 30 days. Data are presented as means ± SD (*n* = 3); different letters indicate a statistically significant difference as determined by an independent samples *t*‐test (*p* < 0.05). (b) SEM images showing the surface morphology of the PLA coupon after 30 days of degradation in the optimized medium (magnification, 5000×).

The influence of the four independent variables on the growth of *P*. *aryabhattai* SNRUSAC3 was visualized through 3D response surface and contour plots generated from the predictive model (Equation 2). These plots depict the relationship between two factors, while the other two are maintained at their central values (Figure 6). The plots revealed that increasing concentrations of glucose (up to 9 g/L), yeast extract (up to 11 g/L), and FeSO_4_ (up to 0.5 g/L) all positively correlated with increased cell density. The optimal ranges were graphically estimated to be 7–9 g/L for glucose (Figures 6a, 6b, and 6c), 7–11 g/L for yeast extract (Figures 6a, 6d, and 6e), and 0.3–0.5 g/L for FeSO_4_ (Figures 6c, 6e, and 6f). In contrast, (NH_4_)_2_SO_4_ exhibited a clear optimal range around 0.5–1.5 g/L (Figures 6b, 6d, and 6f). Numerical optimization of the model predicted that a maximum OD_600_ of 20.628 could be achieved with a medium composition of 7.32 g/L glucose, 8.74 g/L yeast extract, 0.58 g/L (NH_4_)_2_SO_4_, and 0.46 g/L FeSO_4_.

Figure 63D response surface and corresponding contour plots showing the interaction effects of medium components on the growth (OD_600_) of *P. aryabhattai* SNRUSAC3. The interaction between (a) glucose and yeast extract, (b) glucose and (NH_4_)_2_SO_4_, (c) glucose and FeSO_4_, (d) yeast extract and (NH_4_)_2_SO_4_, (e) yeast extract and FeSO_4_, and (f) (NH_4_)_2_SO_4_ and FeSO_4_.

**Figure:**
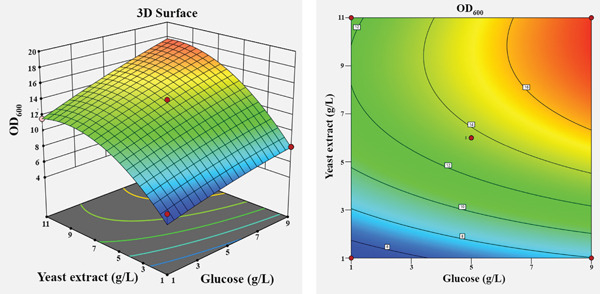
(a)

**Figure:**
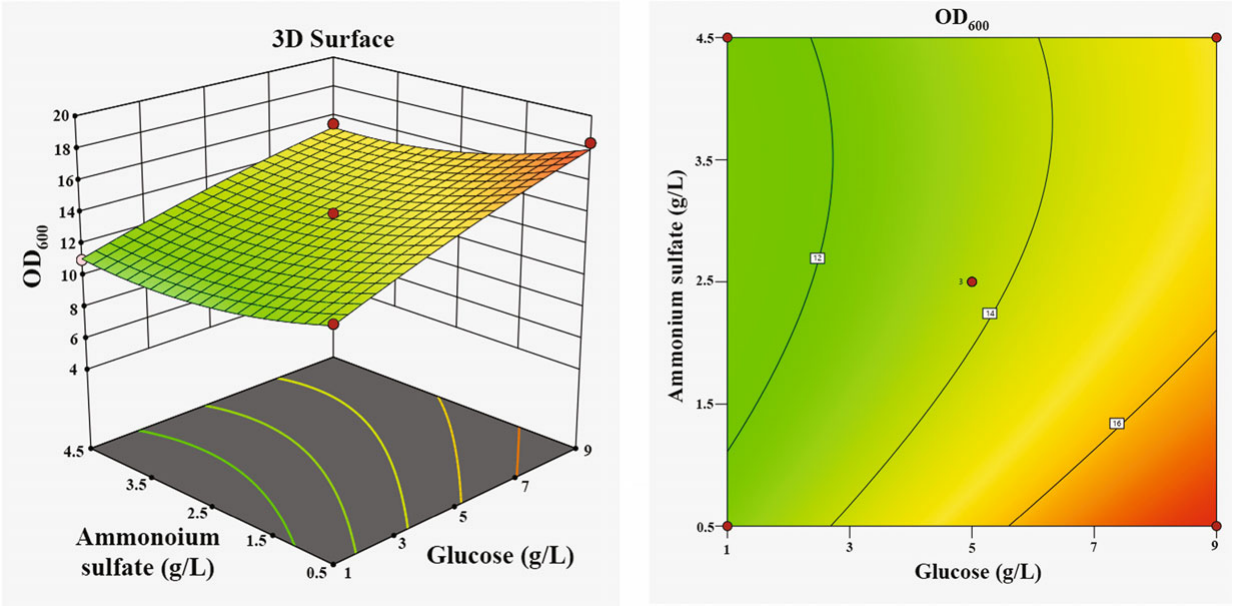
(b)

**Figure:**
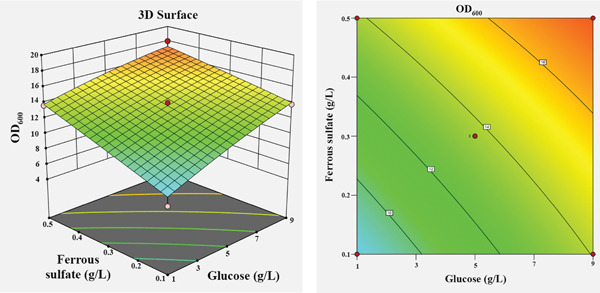
(c)

**Figure:**
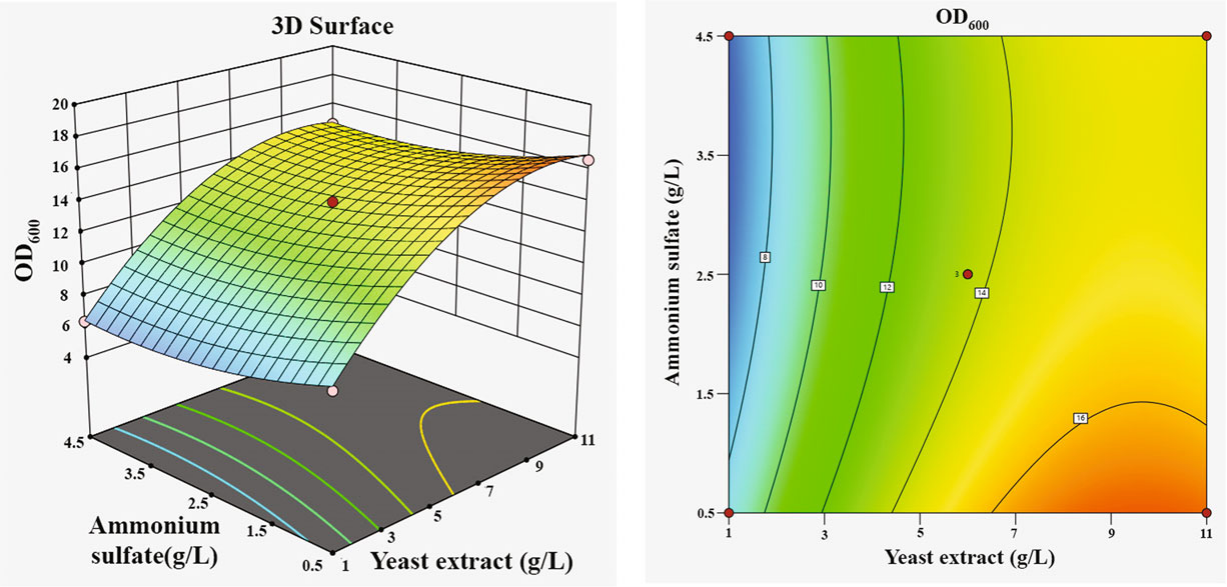
(d)

**Figure:**
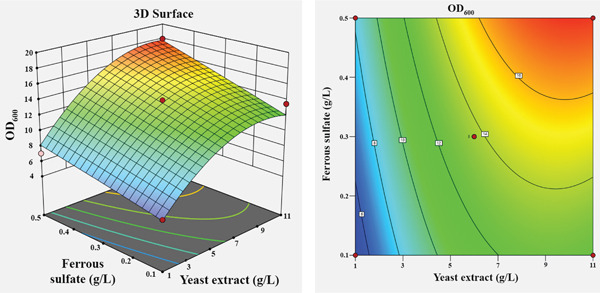
(e)

**Figure:**
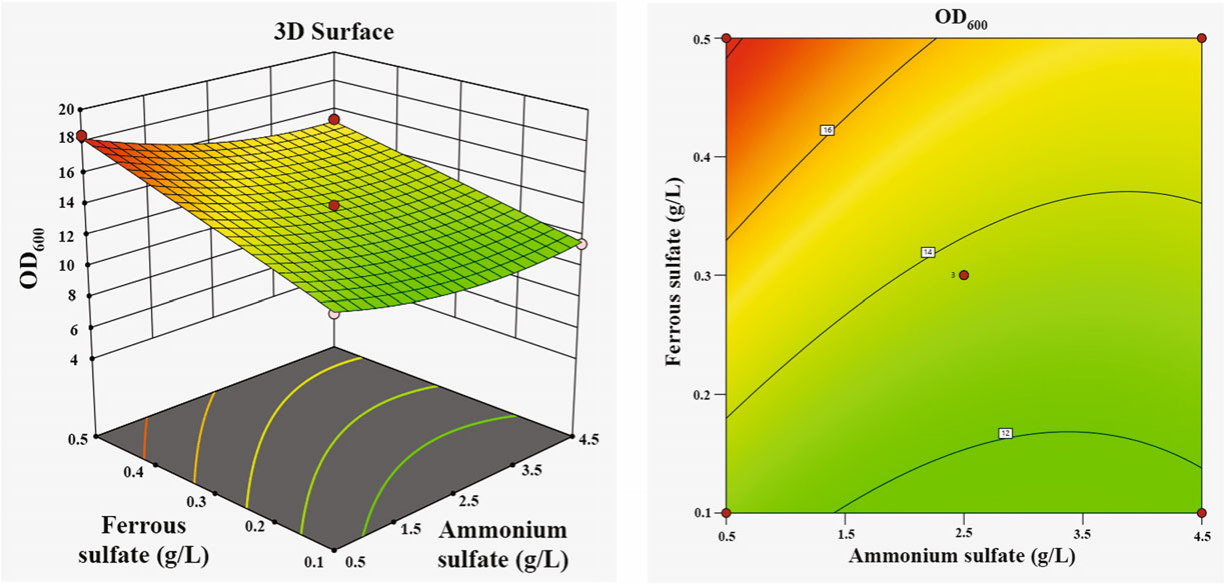
(f)

A confirmation experiment was conducted to validate the RSM model. *P*. *aryabhattai* SNRUSAC3 was cultivated for 24 h in the optimized medium, containing 7.32 g/L of glucose, 8.74 g/L of yeast extract, 0.58 g/L of (NH_4_)_2_SO_4_, and 0.46 g/L of FeSO_4_, along with PLA coupons. The mean experimental OD_600_ was found to be 20.493 ± 0.400. This result showed excellent agreement with the predicted value of 20.628 and fell well within the 95% PI of 18.16–23.09. Furthermore, the percentage error between the observed and predicted values was calculated to be only 0.65%. These findings collectively validate the robustness and high accuracy of the RSM model.

The optimization of medium components is a critical strategy for enhancing microbial growth and, consequently, their degradative capabilities [19, 20]. A significant enhancement in PLA plastic waste degradation was also observed with *P*. *aryabhattai* SNRUSAC3 in the optimized medium. After only 30 days in the optimized medium, the percentage weight loss of the PLA coupons reached 57.61*%* ± 0.04*%*, a substantial increase compared to the 13.16*%* ± 0.06*%* loss observed after a much longer 56‐day period in the BM (Figure 7a). This enhanced degradation was corroborated by SEM analysis, which revealed the extensive erosion of the PLA surface after 30 days. The micrograph showed the formation of numerous large cavities, creating a highly porous, sponge‐like network that extended deep into the material′s inner layers (Figure 7b).

The successful degradation of PLA by *P. aryabhattai* SNRUSAC3 represents a significant finding, as this study is the first to report the PLA‐degrading capability of this species. This discovery expands the known substrate range of *P. aryabhattai*, which has been previously documented for its ability to degrade other challenging polymers, including PET [67, 68], ABS [69], and various PHAs [70]. A critical review of this existing literature, however, reveals that while optimization studies have primarily focused on physical conditions and macronutrients, such as carbon and nitrogen sources, the specific role of the mineral salt component, particularly micronutrients like FeSO_4_, has been largely overlooked. This gap is particularly significant given that our experimental design deliberately included factors from each of these distinct nutritional categories, allowing for a more comprehensive understanding of the nutritional requirements for this species. This highlights the significance of our findings, where the dramatic enhancement in PLA degradation was achieved after a nutritional optimization that identified FeSO_4_ as a novel and key factor. These findings not only establish the importance of iron for PLA degradation by this strain but also suggest its potential role in enhancing the degradation of other plastics. While this nutritional optimization proved highly effective, it is also acknowledged that physical conditions are critical factors for the degradation of other plastics by *P. aryabhattai* [67, 69] and for PLA degradation in other microbial systems [19, 20, 28]. Future investigations integrating the optimization of both physical and nutritional factors are warranted to further enhance degradation. Ultimately, these findings collectively establish *P. aryabhattai* SNRUSAC3 as a highly promising and robust candidate for future applications. Such applications include implementation in larger scale or composting systems, where its high efficiency could be leveraged. However, future studies must also address potential real‐world challenges, such as maintaining microbial stability and biodegradation performance within competitive, nonsterile mixed plastic waste environments.

## 4. Conclusions

This study successfully isolated and identified two highly effective PLA‐degrading bacterial strains, *Bacillus* sp. SNRUSAC1 and *P. aryabhattai* SNRUSAC3, from a compost environment. A key finding of this work is the first‐ever report of PLA degradation by the species *P. aryabhattai*, expanding its known substrate range, which was previously limited to polymers of PET, ABS, and PHAs. Following the optimization of nutritional components, the degradation efficiency of postconsumer PLA packaging waste was dramatically enhanced, with both strains achieving over a fourfold increase in degradation in approximately half the time. Furthermore, a novel, strain‐specific nutritional requirement was revealed, with FeSO_4_ identified as a critical and previously overlooked factor specifically enhancing the growth and degradative activity of *P. aryabhattai*.

Beyond their degradative capabilities, the isolation of these strains from the competitive microbial environment of compost suggests a high degree of robustness. This shared origin provides a strong rationale for investigating other valuable traits, such as plant growth‐promoting and antagonistic properties, to fully assess their potential for development as inoculants in composting and biofertilizer applications. Therefore, future investigations should focus not only on validating their degradation performance in field‐scale composting systems but also on characterizing these additional value‐added traits. The integration of these highly efficient PLA‐degrading strains as inoculants in composting processes could represent a significant step toward creating a closed‐loop system for managing bioplastic waste, thereby contributing to the development of sustainable circular waste management systems.

## Disclosure

The funder had no role in study design, data collection and analysis, decision to publish, or preparation of the manuscript. Both authors read and approved the final version of the manuscript.

## Conflicts of Interest

The authors declare no conflicts of interest.

## Author Contributions

Suwapha Sawiphak and Aroon Wongjiratthiti conceptualized the study, designed and performed the experiments, analyzed the data, and wrote the manuscript.

## Funding

This study was funded by the Sakon Nakhon Rajabhat University (10.13039/100019359) (5/2566).

## Supporting information


**Supporting Information** Additional supporting information can be found online in the Supporting Information section. Figure S1: Qualitative extracellular enzyme assays for strains SNRUSAC1 and SNRUSAC3. Figure S2: Colony morphology, cell morphology, and biochemical characteristics of strain SNRUSAC1. Figure S3: Colony morphology, cell morphology, and biochemical characteristics of strain SNRUSAC3. Table S1: Factors and levels in the PBD. Table S2: PBD matrix of 13 factors with 20 experimental runs and OD_600_ results for *Bacillus* sp. SNRUSAC1 and *P*. *aryabhattai* SNRUSAC3. Table S3: Comparison of SNRUSAC1 nucleotide sequences with reference strains. Table S4: Comparison of SNRUSAC3 nucleotide sequences with reference strains. Table S5: BBD matrix and the experimental results for growth optimization of *Bacillus* sp. SNRUSAC1. Table S6: BBD matrix and the experimental results for growth optimization of *P*. *aryabhattai* SNRUSAC3.

## Data Availability

The gene sequence data generated during this study are available in the GenBank database under Accession Numbers PQ409455 for *Bacillus* sp. SNRUSAC1 and PQ409490 for *Priestia aryabhattai* SNRUSAC3. The remaining data are available from the corresponding author upon reasonable request.
