# Supplementary material for: Highly Efficient Biodegradation of Postconsumer Polylactic Acid Waste: The First Report on Priestia aryabhattai SNRUSAC3 and a Newly Isolated Bacillus sp. SNRUSAC1
Source: Int J Microbiol. 2026 Jan 7;2026:8029640. doi: 10.1155/ijm/8029640 (PMC12780350; doi:10.1155/ijm/8029640)
Supplement: Supplementary file 1 — Supporting Information Additional supporting information can be found online in the Supporting Information section. Figure S1: Qualitative extracellular enzyme assays for strains SNRUSAC1 and SNRUSAC3. Figure S2: Colony morphology, cell morphology, and biochemical characteristics of strain SNRUSAC1. Figure S3: Colony morphology, cell morphology, and biochemical characteristics of strain SNRUSAC3. Table S1: Factors and levels in the PBD. Table S2: PBD matrix of 13 factors with 20 experimental runs and OD600 results for Bacillus sp. SNRUSAC1 and P. aryabhattai SNRUSAC3. Table S3: Comparison of SNRUSAC1 nucleotide sequences with reference strains. Table S4: Comparison of SNRUSAC3 nucleotide sequences with reference strains. Table S5: BBD matrix and the experimental results for growth optimization of Bacillus sp. SNRUSAC1. Table S6: BBD matrix and the experimental results for growth optimization of P. aryabhattai SNRUSAC3. [file IJM-2026-8029640-s001.docx]

**Supplementary Material for**

Highly Efficient Biodegradation of Post-Consumer Polylactic Acid Waste: The First Report on *Priestia aryabhattai* SNRUSAC3 and a Newly Isolated *Bacillus* sp. SNRUSAC1

Suwapha Sawiphak ^a^, Aroon Wongjiratthiti ^a, *^

^a^ Program of Biology, Faculty of Science and Technology,

Sakon Nakhon Rajabhat University, Sakon Nakhon, 47000, Thailand

*Corresponding author: mic_610@snru.ac.th

**Fig. S1.** Qualitative extracellular enzyme assays for strains SNRUSAC1 and SNRUSAC3: (a) Esterase/lipase activity of SNRUSAC1 on Tributyrin Agar. (b) Protease activity of SNRUSAC1 on Skim Milk Agar. (c) Esterase/lipase activity of SNRUSAC3 on Tributyrin Agar. (d) Protease activity of SNRUSAC3 on Skim Milk Agar. Enzymatic activity is indicated by the formation of a clear hydrolysis zone after incubation at 37 °C for 2 days.

**Fig. S2.** Colony morphology, cell morphology, and biochemical characteristics of strain SNRUSAC1: (a) Colony morphology after 18 h incubation on NA at 37 °C; (b) Cell morphology observed under a phase-contrast microscope at 1000× magnification after 18 h incubation at 37 °C; (c) Gram reaction and biochemical characteristics.

**Fig. S3.** Colony morphology, cell morphology, and biochemical characteristics of strain SNRUSAC3: (a) Colony morphology after 18 h incubation on NA at 37 °C; (b) Cell morphology observed under a phase-contrast microscope at 1000× magnification after 18 h incubation at 37 °C; (c) Gram reaction and biochemical characteristics.

**Table S1**

Factors and levels in the PBD.

**Table S2**

PBD matrix of 13 factors with 20 experimental runs and OD_600_ results for *Bacillus* sp. SNRUSAC1 and *P*. *aryabhattai* SNRUSAC3.

**Table S3**

Comparison of SNRUSAC1 nucleotide sequences with reference strains.

**Table S4**

Comparison of SNRUSAC3 nucleotide sequences with reference strains.

**Table S5**

BBD matrix and the experimental results for growth optimization of *Bacillus* sp. SNRUSAC1.

**Table S6**

BBD matrix and the experimental results for growth optimization of *P*. *aryabhattai* SNRUSAC3.


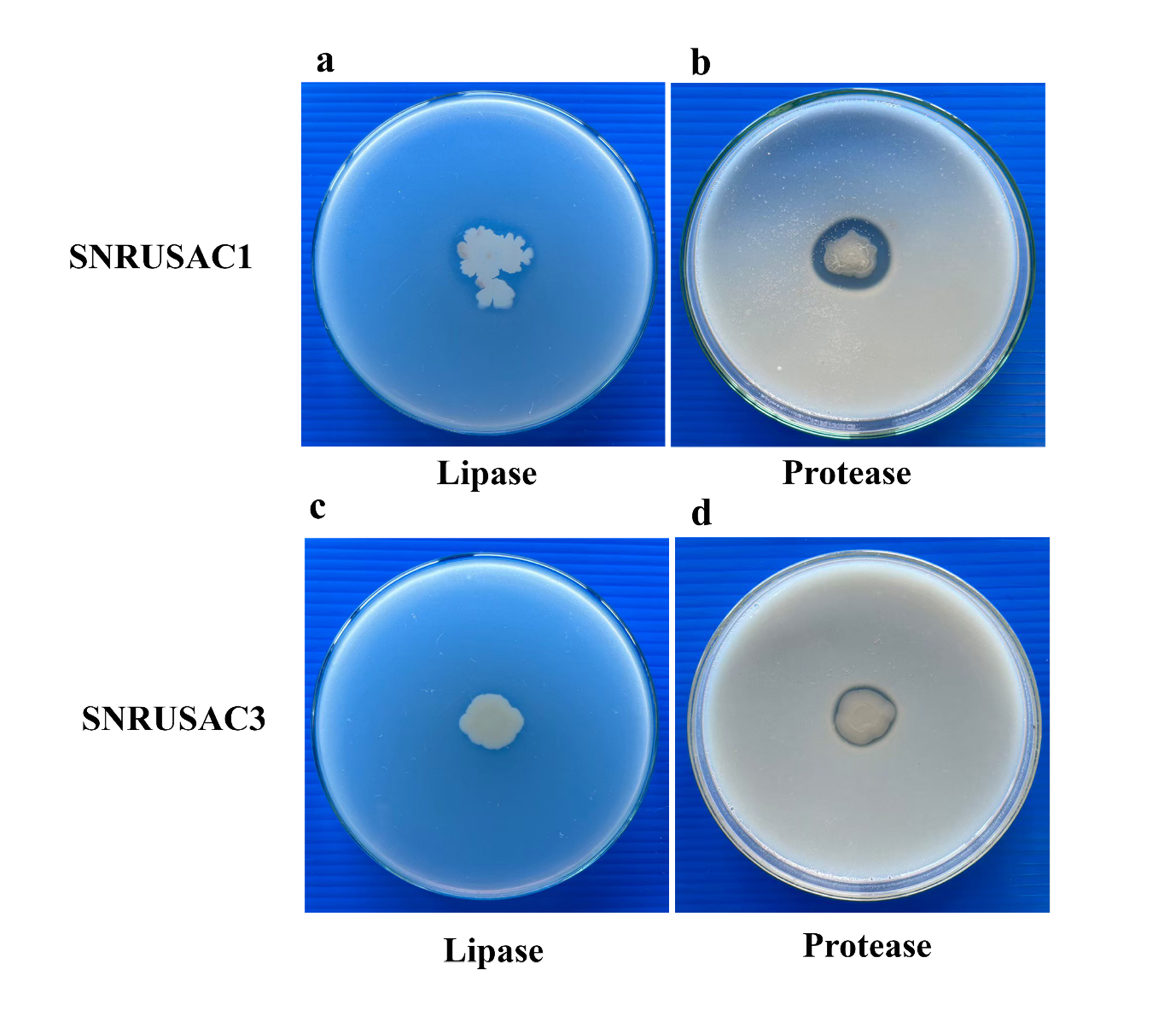


**Fig. S1.** Qualitative extracellular enzyme assays for strains SNRUSAC1 and SNRUSAC3: (a) Esterase/lipase activity of SNRUSAC1 on Tributyrin Agar. (b) Protease activity of SNRUSAC1 on Skim Milk Agar. (c) Esterase/lipase activity of SNRUSAC3 on Tributyrin Agar. (d) Protease activity of SNRUSAC3 on Skim Milk Agar. Enzymatic activity is indicated by the formation of a clear hydrolysis zone after incubation at 37 °C for 2 days.


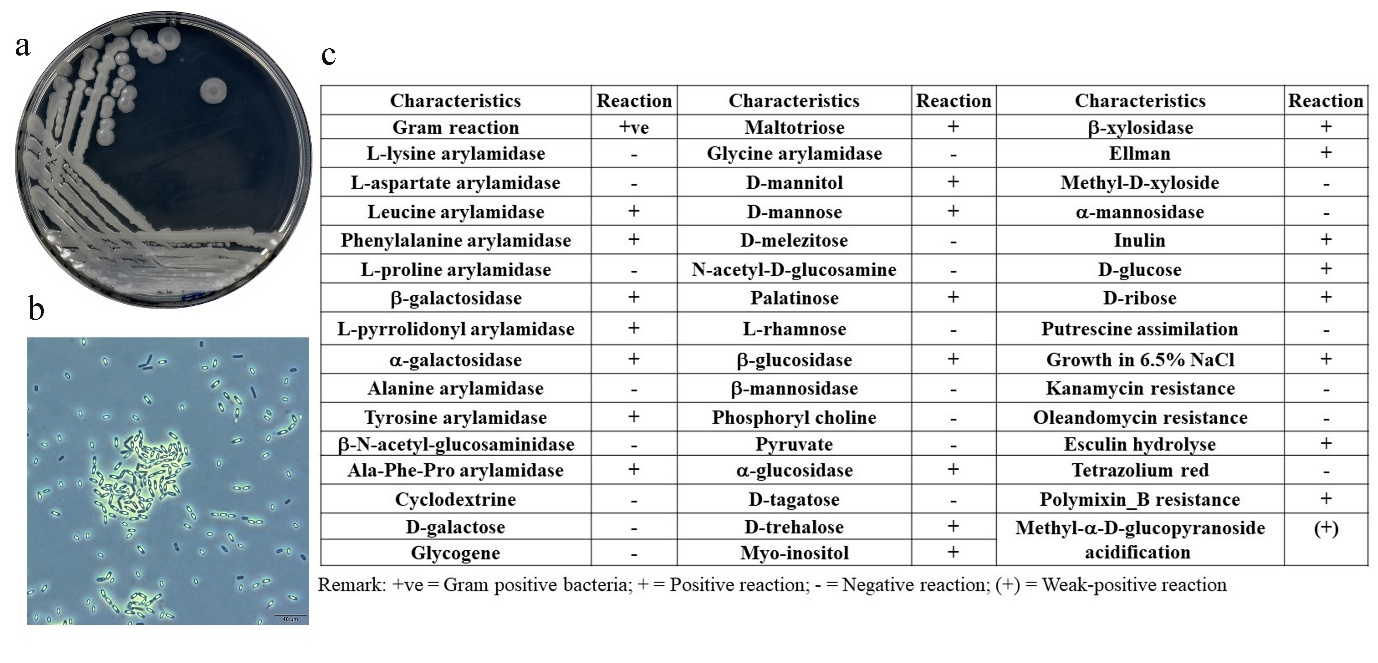


**Fig. S2.** Colony morphology, cell morphology, and biochemical characteristics of strain SNRUSAC1: (a) Colony morphology after 18 h incubation on NA at 37 °C; (b) Cell morphology observed under a phase-contrast microscope at 1000× magnification after 18 h incubation at 37 °C; (c) Gram reaction and biochemical characteristics.


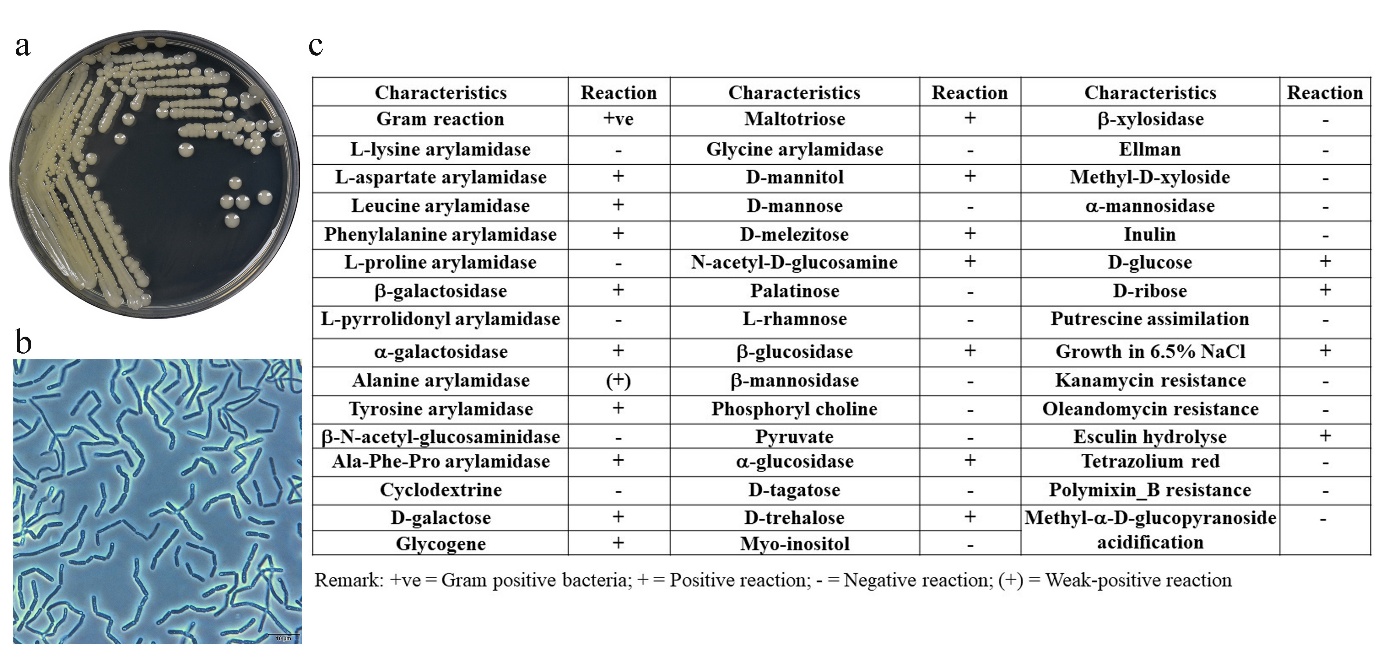


**Fig. S3.** Colony morphology, cell morphology, and biochemical characteristics of strain SNRUSAC3: (a) Colony morphology after 18 h incubation on NA at 37 °C; (b) Cell morphology observed under a phase-contrast microscope at 1000× magnification after 18 h incubation at 37 °C; (c) Gram reaction and biochemical characteristics.

**Table S1**

Factors and levels in the PBD.

| Factors (g/L) | Symbols | High level (+1) | Low level (-1) |
| --- | --- | --- | --- |
| K_2_HPO_4_ | (*X_1_*) | 3 | 1 |
| KH_2_PO_4_ | (*X_2_*) | 3 | 1 |
| MgSO_4_·7H_2_O | (*X_3_*) | 0.7 | 0.3 |
| (NH_4_)_2_SO_4_ | (*X_4_*) | 6 | 2 |
| glucose | (*X_5_*) | 5 | 1 |
| sucrose | (*X_6_*) | 5 | 1 |
| tryptone | (*X_7_*) | 5 | 1 |
| beef extract | (*X_8_*) | 5 | 1 |
| gelatin | (*X_9_*) | 5 | 1 |
| peptone | (*X_10_*) | 5 | 1 |
| yeast extract | (*X_11_*) | 5 | 1 |
| FeSO_4_ | (*X_12_*) | 0.02 | 0 |
| NaCl | (*X_13_*) | 5 | 0 |

**Table S2**

PBD matrix of 13 factors with 20 experimental runs and OD_600_ results for *Bacillus* sp. SNRUSAC1 and *P*. *aryabhattai* SNRUSAC3.

| Run | *X_1_* | *X_2_* | *X_3_* | *X_4_* | *X_5_* | *X_6_* | *X_7_* | *X_8_* | *X_9_* | *X_10_* | *X_11_* | *X_12_* | *X_13_* | OD_600_ | |
| --- | --- | --- | --- | --- | --- | --- | --- | --- | --- | --- | --- | --- | --- | --- | --- |
|  |  |  |  |  |  |  |  |  |  |  |  |  |  | *Bacillus* sp. SNRUSAC1 | *P*. *aryabhattai* SNRUSAC3 |
| 1 | +1 | -1 | -1 | +1 | +1 | +1 | +1 | -1 | +1 | -1 | +1 | -1 | -1 | 4.767 | 11.072 |
| 2 | +1 | +1 | -1 | +1 | -1 | +1 | -1 | -1 | -1 | -1 | +1 | +1 | -1 | 4.200 | 9.750 |
| 3 | -1 | +1 | +1 | +1 | +1 | -1 | +1 | -1 | +1 | -1 | -1 | -1 | -1 | 3.805 | 9.130 |
| 4 | -1 | +1 | +1 | -1 | -1 | +1 | +1 | +1 | +1 | -1 | +1 | -1 | +1 | 6.965 | 12.007 |
| 5 | +1 | +1 | -1 | +1 | +1 | -1 | -1 | +1 | +1 | +1 | +1 | -1 | +1 | 5.873 | 11.970 |
| 6 | -1 | +1 | +1 | -1 | +1 | +1 | -1 | -1 | +1 | +1 | +1 | +1 | -1 | 3.945 | 15.975 |
| 7 | -1 | -1 | +1 | +1 | -1 | +1 | +1 | -1 | -1 | +1 | +1 | +1 | +1 | 6.212 | 13.311 |
| 8 | +1 | -1 | +1 | -1 | +1 | -1 | -1 | -1 | -1 | +1 | +1 | -1 | +1 | 4.260 | 13.191 |
| 9 | +1 | +1 | -1 | -1 | +1 | +1 | +1 | +1 | -1 | +1 | -1 | +1 | -1 | 4.333 | 17.612 |
| 10 | +1 | -1 | +1 | +1 | -1 | -1 | +1 | +1 | +1 | +1 | -1 | +1 | -1 | 3.231 | 10.073 |
| 11 | -1 | +1 | -1 | +1 | -1 | -1 | -1 | -1 | +1 | +1 | -1 | +1 | +1 | 2.244 | 7.511 |
| 12 | -1 | -1 | +1 | +1 | +1 | +1 | -1 | +1 | -1 | +1 | -1 | -1 | -1 | 3.248 | 7.234 |
| 13 | +1 | -1 | -1 | -1 | -1 | +1 | +1 | -1 | +1 | +1 | -1 | -1 | +1 | 3.998 | 7.261 |
| 14 | -1 | -1 | -1 | +1 | +1 | -1 | +1 | +1 | -1 | -1 | +1 | +1 | +1 | 5.957 | 13.829 |
| 15 | +1 | +1 | +1 | -1 | +1 | -1 | +1 | -1 | -1 | -1 | -1 | +1 | +1 | 3.301 | 13.126 |
| 16 | -1 | +1 | -1 | -1 | -1 | -1 | +1 | +1 | -1 | +1 | +1 | -1 | -1 | 3.406 | 10.055 |
| 17 | -1 | -1 | -1 | -1 | +1 | +1 | -1 | +1 | +1 | -1 | -1 | +1 | +1 | 3.843 | 15.808 |
| 18 | +1 | -1 | +1 | -1 | -1 | -1 | -1 | +1 | +1 | -1 | +1 | +1 | -1 | 2.643 | 11.331 |
| 19 | -1 | -1 | -1 | -1 | -1 | -1 | -1 | -1 | -1 | -1 | -1 | -1 | -1 | 1.312 | 5.559 |
| 20 | +1 | +1 | +1 | +1 | -1 | +1 | -1 | +1 | -1 | -1 | -1 | -1 | +1 | 3.280 | 6.910 |

**Table S3**

Comparison of SNRUSAC1 nucleotide sequences with reference strains.

| Rank | Name | Strain | Accession | Pairwise Similarity (%) | Mismatch  /Total nt |
| --- | --- | --- | --- | --- | --- |
| 1 | *Bacillus tequilensis* | KCTC 13622 | AYTO01000043 | 99.93 | 1/1444 |
| 2 | *Bacillus cabrialesii* | TE3 | MK462260 | 99.93 | 1/1444 |
| 3 | *Bacillus inaquosorum* | KCTC 13429 | AMXN01000021 | 99.93 | 1/1444 |
| 4 | *Bacillus subtilis* | NCIB 3610 | ABQL01000001 | 99.86 | 2/1444 |
| 5 | *Bacillus stercoris* | JCM 30051 | MN536904 | 99.79 | 3/1444 |
| 6 | *Bacillus spizizenii* | NRRL B-23049 | CP002905 | 99.79 | 3/1444 |
| 7 | *Bacillus velezensis* | CR-502 | AY603658 | 99.71 | 4/1403 |
| 8 | *Bacillus halotolerans* | ATCC 25096 | LPVF01000003 | 99.65 | 5/1444 |
| 9 | *Bacillus mojavensis* | RO-H-1 | JH600280 | 99.58 | 6/1444 |
| 10 | *Bacillus vallismortis* | DV1-F-3 | JH600273 | 99.58 | 6/1444 |
| 11 | *Bacillus nakamurai* | NRRL B-41091 | LSAZ01000028 | 99.58 | 6/1444 |
| 12 | *Bacillus siamensis* | KCTC 13613 | AJVF01000043 | 99.45 | 8/1444 |
| 13 | *Bacillus amyloliquefaciens* | DSM 7 | FN597644 | 99.31 | 10/1444 |
| 14 | *Bacillus atrophaeus* | JCM 9070 | AB021181 | 99.24 | 11/1444 |
| 15 | *Bacillus glycinifermentans* | GO-13 | LECW01000063 | 98.75 | 18/1443 |
| 16 | *Bacillus paralicheniformis* | KJ-16 | KY694465 | 98.68 | 19/1443 |
| 17 | *Bacillus haynesii* | NRRL B-41327 | MRBL01000076 | 98.41 | 23/1443 |
| 18 | *Bacillus licheniformis* | ATCC 14580 | AE017333 | 98.34 | 24/1443 |
| 19 | *Bacillus sonorensis* | NBRC 101234 | AYTN01000016 | 98.13 | 27/1443 |
| 20 | *Bacillus swezeyi* | NRRL B-41294 | MRBK01000096 | 98.13 | 27/1443 |
| 21 | *Bacillus aerius* | 24K | AJ831843 | 97.78 | 32/1441 |
| 22 | *Bacillus altitudinis* | 41KF2b | ASJC01000029 | 97.16 | 41/1443 |
| 23 | *Bacillus xiamenensis* | HYC-10 | AMSH01000114 | 97.09 | 42/1443 |
| 24 | *Bacillus safensis* subsp. *safensis* | FO-36b | ASJD01000027 | 96.95 | 44/1443 |
| 25 | *Bacillus safensis* subsp. *osmophilus* | BC09 | KY990920 | 96.95 | 44/1443 |
| 26 | *Bacillus pumilus* | ATCC 7061 | ABRX01000007 | 96.88 | 45/1443 |
| 27 | *Bacillus zhangzhouensis* | DW5-4 | JOTP01000061 | 96.88 | 45/1443 |
| 28 | *Bacillus australimaris* | NH7I_1 | JX680098 | 96.81 | 46/1443 |
| 29 | *Rossellomorea oryzaecorticis* | R1 | KF548480 | 96.43 | 39/1091 |
| 30 | *Bacillus salacetis* | SKP7-4 | LC367333 | 96.36 | 52/1427 |

**Table S4**

Comparison of SNRUSAC3 nucleotide sequences with reference strains.

| Rank | Name | Strain | Accession | Pairwise Similarity (%) | Mismatch  /Total nt |
| --- | --- | --- | --- | --- | --- |
| 1 | *Priestia aryabhattai* | B8W22 | EF114313 | 100.00 | 0/1474 |
| 2 | *Priestia megaterium* | NBRC 15308 | JJMH01000057 | 99.86 | 2/1474 |
| 3 | *Priestia flexa* | NBRC 15715 | BCVD01000224 | 98.85 | 17/1474 |
| 4 | *Bacillus pseudoflexus* | RC1 | FN999944 | 98.28 | 25/1451 |
| 5 | *Priestia qingshengii* | G19 | JX293295 | 98.24 | 25/1420 |
| 6 | *Priestia paraflexa* | RC2 | FN999943 | 97.83 | 30/1381 |
| 7 | *Metabacillus iocasae* | S36 | KY462210 | 96.46 | 52/1471 |
| 8 | *Sutcliffiella cohnii* | NBRC 15565 | BCUW01000190 | 96.13 | 57/1474 |
| 9 | *Falsibacillus pallidus* | DSM 25281 | QQAY01000036 | 96.13 | 57/1472 |
| 10 | *Neobacillus pocheonensis* | Gsoil 420 | AB245377 | 96.13 | 57/1472 |
| 11 | *Priestia koreensis* | DSM 16467 | LILC01000014 | 96.00 | 59/1474 |
| 12 | *Cytobacillus oceanisediminis* | H2 | GQ292772 | 95.91 | 57/1393 |
| 13 | *Metabacillus herbersteinensis* | D-1-5a | AJ781029 | 95.90 | 60/1462 |
| 14 | *Peribacillus huizhouensis* | GSS03 | KJ464756 | 95.89 | 60/1459 |
| 15 | *Cytobacillus depressus* | BZ1 | KP259553 | 95.86 | 60/1449 |
| 16 | *Neobacillus bataviensis* | LMG 21833 | AJ542508 | 95.86 | 61/1472 |
| 17 | *Peribacillus asahii* | MA001 | QWVS01000027 | 95.86 | 61/1472 |
| 18 | *Niallia circulans* | ATCC 4513 | AY724690 | 95.85 | 61/1469 |
| 19 | *Neobacillus cucumis* | AP-6 | KT895286 | 95.80 | 61/1451 |
| 20 | *Metabacillus idriensis* | SMC 4352-2 | AY904033 | 95.79 | 60/1425 |
| 21 | *Niallia oryzisoli* | 1DS3-10 | KT886063 | 95.79 | 62/1472 |
| 22 | *Robertmurraya kyonggiensis* | NB22 | JF896450 | 95.73 | 62/1452 |
| 23 | *Neobacillus soli* | NBRC 102451 | BCVI01000121 | 95.72 | 63/1472 |
| 24 | *Cytobacillus kochii* | WCC 4582 | FN995265 | 95.70 | 63/1466 |
| 25 | *Cytobacillus purgationiresistens* | DS22 | FR666703 | 95.68 | 62/1435 |
| 26 | *Neobacillus drentensis* | LMG 21831 | AJ542506 | 95.67 | 61/1408 |
| 27 | *Bacillus tianshenii* | YIM M13235 | KF811034 | 95.65 | 64/1472 |
| 28 | *Peribacillus butanolivorans* | DSM 18926 | LGYA01000001 | 95.65 | 64/1472 |
| 29 | *Sutcliffiella halmapala* | DSM 8723 | KV917375 | 95.65 | 64/1472 |
| 30 | *Cytobacillus eiseniae* | A1-2 | HM035089 | 95.65 | 64/1472 |

**Table S5**

BBD matrix and the experimental results for growth optimization of *Bacillus* sp. SNRUSAC1.

| Run | *X_1_*: Yeast extract  (g/L) | *X_2_*: Tryptone (g/L) | *X_3_*:  NaCl  (g/L) | *X_4_*:  Sucrose  (g/L) | OD_600_ | |
| --- | --- | --- | --- | --- | --- | --- |
|  |  |  |  |  | Actual | Predicted |
| 1 | 13 | 6 | 1 | 5 | 7.140 | 7.031 |
| 2 | 7 | 1 | 1 | 5 | 4.950 | 4.656 |
| 3 | 1 | 1 | 5 | 5 | 1.356 | 1.741 |
| 4 | 13 | 6 | 9 | 5 | 6.691 | 6.489 |
| 5 | 7 | 11 | 5 | 1 | 5.705 | 5.549 |
| 6 | 7 | 6 | 9 | 9 | 5.180 | 5.324 |
| 7 | 7 | 6 | 1 | 9 | 5.273 | 5.428 |
| 8 | 13 | 11 | 5 | 5 | 7.200 | 7.033 |
| 9 | 1 | 6 | 5 | 9 | 2.669 | 2.590 |
| 10 | 7 | 11 | 1 | 5 | 5.390 | 5.624 |
| 11 | 7 | 11 | 9 | 5 | 4.988 | 5.327 |
| 12 | 7 | 1 | 5 | 1 | 4.150 | 4.099 |
| 13 | 7 | 11 | 5 | 9 | 5.604 | 5.391 |
| 14 | 7 | 6 | 1 | 1 | 5.030 | 5.103 |
| 15 | 7 | 6 | 5 | 5 | 6.002 | 5.806 |
| 16 | 1 | 11 | 5 | 5 | 2.712 | 2.674 |
| 17 | 13 | 1 | 5 | 5 | 5.968 | 6.224 |
| 18 | 1 | 6 | 1 | 5 | 2.328 | 2.267 |
| 19 | 7 | 1 | 9 | 5 | 4.742 | 4.554 |
| 20 | 7 | 1 | 5 | 9 | 5.206 | 5.099 |
| 21 | 1 | 6 | 9 | 5 | 2.564 | 2.410 |
| 22 | 13 | 6 | 5 | 9 | 6.820 | 6.918 |
| 23 | 7 | 6 | 5 | 5 | 5.600 | 5.806 |
| 24 | 13 | 6 | 5 | 1 | 6.466 | 6.590 |
| 25 | 1 | 6 | 5 | 1 | 2.129 | 2.076 |
| 26 | 7 | 6 | 9 | 1 | 4.746 | 4.808 |
| 27 | 7 | 6 | 5 | 5 | 5.815 | 5.806 |

**Table S6**

BBD matrix and the experimental results for growth optimization of *P*. *aryabhattai* SNRUSAC3.

| Run | *X_1_*:  Glucose (g/L) | *X_2_*: Yeast extract (g/L) | *X_3_*:  (NH_4_)_2_SO_4_  (g/L) | *X_4_*:  FeSO_4_  (g/L) | OD_600_ | |
| --- | --- | --- | --- | --- | --- | --- |
|  |  |  |  |  | Actual | Predicted |
| 1 | 5 | 1 | 2.5 | 0.1 | 5.075 | 4.961 |
| 2 | 5 | 11 | 2.5 | 0.5 | 17.955 | 17.736 |
| 3 | 9 | 6 | 4.5 | 0.3 | 15.461 | 15.218 |
| 4 | 9 | 11 | 2.5 | 0.3 | 16.511 | 17.731 |
| 5 | 5 | 6 | 0.5 | 0.5 | 18.393 | 18.221 |
| 6 | 5 | 11 | 2.5 | 0.1 | 13.545 | 12.106 |
| 7 | 9 | 6 | 0.5 | 0.3 | 18.358 | 17.956 |
| 8 | 5 | 6 | 4.5 | 0.5 | 15.321 | 15.190 |
| 9 | 5 | 1 | 4.5 | 0.3 | 6.361 | 6.432 |
| 10 | 5 | 11 | 4.5 | 0.3 | 14.683 | 14.794 |
| 11 | 5 | 6 | 2.5 | 0.3 | 14.028 | 13.647 |
| 12 | 5 | 1 | 0.5 | 0.3 | 8.321 | 8.591 |
| 13 | 1 | 1 | 2.5 | 0.3 | 5.985 | 4.718 |
| 14 | 5 | 6 | 2.5 | 0.3 | 13.646 | 13.647 |
| 15 | 1 | 6 | 0.5 | 0.3 | 12.793 | 12.701 |
| 16 | 5 | 1 | 2.5 | 0.5 | 7.079 | 8.183 |
| 17 | 9 | 6 | 2.5 | 0.1 | 13.825 | 14.051 |
| 18 | 1 | 6 | 2.5 | 0.1 | 7.035 | 8.154 |
| 19 | 1 | 6 | 2.5 | 0.5 | 13.659 | 13.814 |
| 20 | 5 | 6 | 4.5 | 0.1 | 11.524 | 11.648 |
| 21 | 5 | 6 | 2.5 | 0.3 | 13.268 | 13.647 |
| 22 | 1 | 11 | 2.5 | 0.3 | 11.620 | 11.636 |
| 23 | 5 | 11 | 0.5 | 0.3 | 16.616 | 16.927 |
| 24 | 9 | 1 | 2.5 | 0.3 | 8.015 | 7.951 |
| 25 | 1 | 6 | 4.5 | 0.3 | 11.078 | 11.145 |
| 26 | 9 | 6 | 2.5 | 0.5 | 17.981 | 17.244 |
| 27 | 5 | 6 | 0.5 | 0.1 | 12.828 | 12.911 |
